# Concurrent Host-Pathogen Transcriptional Responses in a *Clostridium perfringens* Murine Myonecrosis Infection

**DOI:** 10.1128/mBio.00473-18

**Published:** 2018-03-27

**Authors:** Lee-Yean Low, Paul F. Harrison, Jodee Gould, David R. Powell, Jocelyn M. Choo, Samuel C. Forster, Ross Chapman, Linden J. Gearing, Jackie K. Cheung, Paul Hertzog, Julian I. Rood

**Affiliations:** a Infection and Immunity Program, Monash Biomedicine Discovery Institute and Department of Microbiology, Monash University, Clayton, Australia; b Monash Bioinformatics Platform, Monash University, Clayton, Australia; c Department of Molecular and Translational Science, Centre for Innate Immunity and Infectious Diseases, Hudson Institute of Medical Research, School of Clinical Science, Monash University, Clayton, Australia; University of Oklahoma Health Sciences Center

**Keywords:** clostridial myonecrosis, *Clostridium perfringens*, RNA-seq, gas gangrene, host-pathogen interactions, inflammasome, innate immunity, transcriptomics

## Abstract

To obtain an insight into host-pathogen interactions in clostridial myonecrosis, we carried out comparative transcriptome analysis of both the bacterium and the host in a murine *Clostridium perfringens* infection model, which is the first time that such an investigation has been conducted. Analysis of the host transcriptome from infected muscle tissues indicated that many genes were upregulated compared to the results seen with mock-infected mice. These genes were enriched for host defense pathways, including Toll-like receptor (TLR) and Nod-like receptor (NLR) signaling components. Real-time PCR confirmed that host TLR2 and NLRP3 inflammasome genes were induced in response to *C. perfringens* infection. Comparison of the transcriptome of *C. perfringens* cells from the infected tissues with that from broth cultures showed that host selective pressure induced a global change in *C. perfringens* gene expression. A total of 33% (923) of *C. perfringens* genes were differentially regulated, including 10 potential virulence genes that were upregulated relative to their expression *in vitro*. These genes encoded putative proteins that may be involved in the synthesis of cell wall-associated macromolecules, in adhesion to host cells, or in protection from host cationic antimicrobial peptides. This report presents the first successful expression profiling of coregulated transcriptomes of bacterial and host genes during a clostridial myonecrosis infection and provides new insights into disease pathogenesis and host-pathogen interactions.

## IMPORTANCE

*Clostridium perfringens* is the causative agent of traumatic clostridial myonecrosis, or gas gangrene. In this study, we carried out transcriptional analysis of both the host and the bacterial pathogen in a mouse myonecrosis infection. The results showed that in comparison to mock-infected control tissues, muscle tissues from *C. perfringens*-infected mice had a significantly altered gene expression profile. In particular, the expression of many genes involved in the innate immune system was upregulated. Comparison of the expression profiles of *C. perfringens* cells isolated from the infected tissues with those from equivalent broth cultures identified many potential virulence genes that were significantly upregulated *in vivo*. These studies have provided a new understanding of the range of factors involved in host-pathogen interactions in a myonecrosis infection.

## INTRODUCTION

*Clostridium perfringens* is a Gram-positive anaerobic rod that causes clostridial myonecrosis, or gas gangrene, a fulminant soft tissue infection that is often fatal (1). The infection frequently starts with the introduction of *C. perfringens* vegetative cells or spores into soft tissues, following a traumatic injury. Impaired blood circulation at the site of infection provides a growth advantage to the anaerobic *C. perfringens* cells, which in turn enhances the progression of myonecrosis (2). The disease is characterized by fever, pain, massive local edema, gas production, and severe muscle tissue destruction, and it often develops into systemic toxemia, shock, sepsis, or death, which occurs in more than 50% of cases (3).

The essential virulence factor in *C. perfringens*-mediated clostridial myonecrosis is alpha-toxin (4, 5), which is encoded by the *plc* (*cpa*) gene and has both phospholipase C and sphingomyelinase activities (6). Perfringolysin O (PFO or θ-toxin), which is a pore-forming toxin encoded by the *pfoA* gene, works synergistically with alpha-toxin in the disease process (5). Using a mouse myonecrosis model, it has been demonstrated that alpha-toxin and PFO upregulate adhesion molecules on the surface of inflammatory cells, thereby enhancing intravascular cell aggregation, which subsequently leads to vascular occlusion (7–10). Furthermore, alpha-toxin and PFO subvert the host immune response by altering the extravasation of inflammatory cells, which limits inflammatory cell infiltration to the site of the infection, a hallmark of clostridial myonecrosis (3, 11, 12).

Although the toxins involved in clostridial myonecrosis have been identified, our understanding of the details of the infection process is still limited. Furthermore, the host response is unusual because it does not appear to include a conventional inflammatory response. Therefore, deciphering the dynamic interactions between pathogen and host is important for elucidating the strategies that *C. perfringens* employs to overcome the host innate immune system and to determine how infected host cells respond to the invading pathogen. As these interactions initiate a series of signaling cascades that lead to altered gene expression in both organisms, we measured the genome-wide transcription of bacterial and host genes in a mouse myonecrosis model. Since clostridial myonecrosis is a fulminant disease that leads to rapid tissue destruction and extensive necrosis, the dual transcriptomic study of pathogen and host was performed at the early stage of infection before the host had succumbed to disease.

*C. perfringens* genes that were differentially regulated in the host environment and host signaling cascades that were altered in response to a *C. perfringens* infection were identified. The results led to the identification of potential new virulence factors of *C. perfringens*, factors that may interact with the host to drive the innate response, resulting in activation of sensors such as the Nod-like receptor P3 (NLRP3) inflammasome and the consequent production of effector cytokines.

## RESULTS AND DISCUSSION

### Concurrent pathogen and host transcriptome changes.

The interaction between the host and the bacterial pathogen during an infection initiates a series of events that alter gene expression in both organisms. To identify novel virulence factors of *C. perfringens in vivo*, and to understand the unique way in which the host responds to this infection, we carried out transcriptional profiling of both the pathogen and the host in infected murine muscle tissues. In this model system, an inoculum of *C. perfringens* cells (approximately 10^9^ CFU) sufficient to induce a typical clostridial myonecrosis pathology was injected into both hind legs of each mouse. To enable direct comparisons of the transcriptomes of *in vivo*- and *in vitro-*derived bacterial cells, the *in vitro* culture inoculum was standardized to the same final concentration as that used with the cells in the animal model.

**FIG 1:**
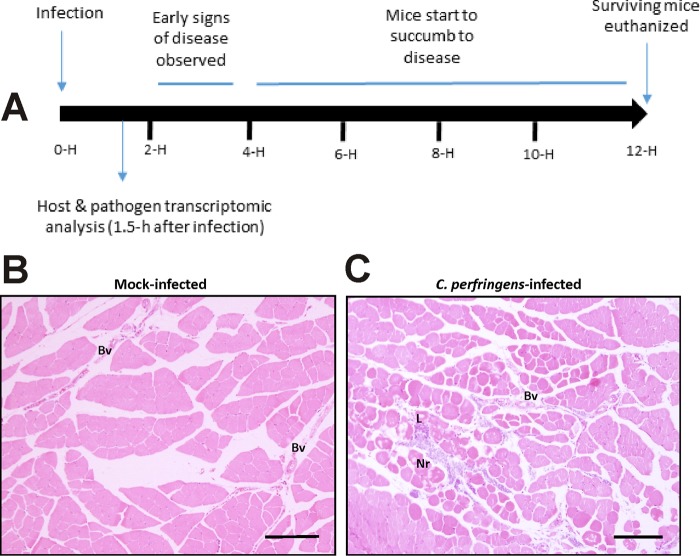
Disease progression in a mouse myonecrosis model. (A) The diagram shows the normal course of disease progression, with the time after infection shown in hours (H). In this study, samples were taken after 1.5 h, before significant clinical signs were observed. (B and C) Mock-infected muscle tissue (B) and *C. perfringens*-infected tissue (C) are shown stained with hematoxylin and eosin 1.5 h after infection. Bars, 10 µm. Bv, blood vessels; Nr, necrotic tissue; L, leukocytes.

**FIG 2:**
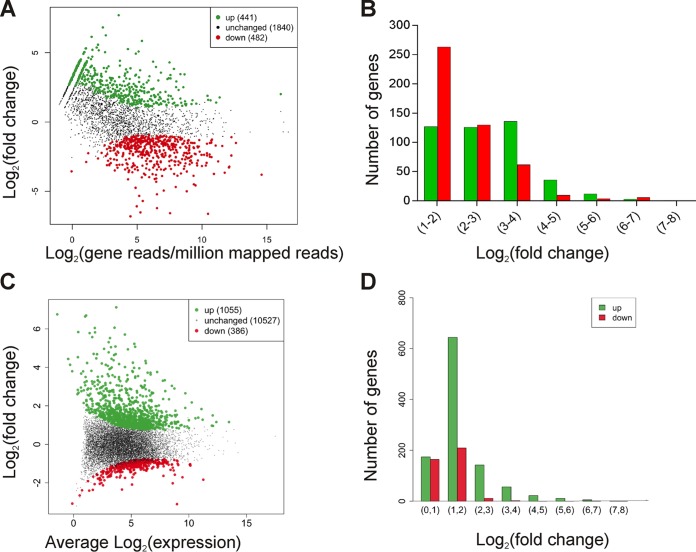
Relative levels of expression of host and *C. perfringens* genes. (A) Differentially expressed genes in *C. perfringens* JIR325 cells cultured *in vivo* compared to *in vitro*, as analyzed by the DESeq R package (87). Each point represents a gene. (B) Proportions of differentially expressed *C. perfringens* genes relative to their altered levels of expression. [FDR, <0.01; log_2_(fold change), >1]. (C) Differential expression of host genes as analyzed by the DESeq R package. Each point represents a separate gene. (D) Proportions of differentially expressed host genes relative to their altered levels of gene expression. [FDR, <0.01; log_2_(fold change), >2]. In all graphs, green dots and bars represent significantly upregulated genes and red dots and bars represent significantly downregulated genes.

Disease progression in this murine model is rapid (Fig. 1A), with signs of disease such as limping and swelling of the footpad and thigh observed as early as 2 h after infection (5, 13). Extensive muscle necrosis develops into severe disease at approximately 4 h postinfection, when mice need to be euthanized for ethical reasons. The RNA used in this study was extracted from mice killed at 1.5 h postinfection, before gross pathological signs of infection such as limping or blackening were observed. Histological analysis revealed that muscle sections from the infected mice were characterized by only limited necrosis of the muscle and very little leukocyte infiltration into the tissues (Fig. 1B and C). On the basis of these observations, we concluded that a snapshot of gene expression signatures of viable cells from the pathogen and the host at this time point would provide valuable information on their interactions or reciprocal regulation at this important early stage of the infection process. In general terms, the transcriptome of *C. perfringens* genes during this *in vivo* infection relative to that of the *in vitro* broth cultures showed for the first time that the host environment induced a global change in the expression of 923 genes (Fig. 2A and B) and that the host muscle tissues responded to *C. perfringens* infection by modifying the expression of 1,441 genes (Fig. 2C and D).

### *C. perfringens* infection regulates the transcription of host innate immune response genes.

**FIG 3:**
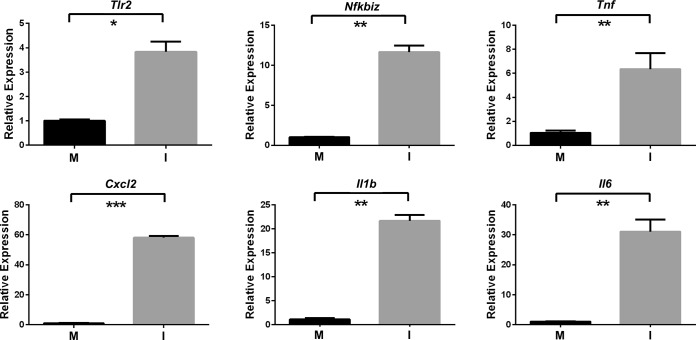
qRT-PCR analysis of selected host transcripts. RNA preparations were from muscle tissues injected with PBS (mock infection; M) or with *C. perfringens* strain JIR325 (infection; I). Values are averages of results from three independent biological replicates ± standard errors of the means. Statistically significant differences (*P* ≤ 0.05 by unpaired *t* test) are denoted by asterisks as follows: *, *P* ≤ 0.05; **, *P* ≤ 0.01; ***, *P* ≤ 0.001.

In response to the *C. perfringens* infection, 1,055 host genes were upregulated and 386 host genes were downregulated (Fig. 2C and D; see also Table S1 in the supplemental material). Specific genes with a range of expression and induction values (Fig. 2D) and in diverse gene ontology (GO) categories were selected for quantitative reverse transcription-PCR (qRT-PCR) validation of the transcriptome sequencing (RNA-seq) data. The transcript levels of the genes encoding Toll-like receptor 2 (TLR2), tumor necrosis factor alpha (TNF-α), NF-κB, interleukin-1 beta (IL1b), IL6, and CXCL2 were significantly increased compared to the control levels, as measured by qRT-PCR (Fig. 3), which was in agreement with the values obtained by RNA-seq (Table S1).

10.1128/mBio.00473-18.4 TABLE S1  Differentially expressed murine genes in muscle tissues from infected mice compared to the mock-infected mice. Download TABLE S1, PDF file, 0.4 MB. Copyright © 2018 Low et al. 2018 Low et al. This content is distributed under the terms of the Creative Commons Attribution 4.0 International license.

Gene set enrichment analyses (GSEA) of the transcriptional changes in host gene expression (Table S2) revealed that the major categories of regulated innate immune response genes included inflammation, inflammasome signaling (encompassing Nod-like receptor P3 [NLRP3] and IL1), and cytokine signaling (inflammation and cell death—IL2, JAK/STAT, IL6, and IL1). Since the unique nature of this infection involves rapid muscle necrosis without substantial inflammation, this analysis suggests that expression of the inflammatory signals did occur, even though a normal inflammatory response was not observed. Other categories of gene expression changes included hypoxia, which was commensurate with the requirements of this anaerobic pathogen; apoptosis (initiating the process); and the expression of adhesion/extracellular matrix (ECM) factors and platelet genes, which was consistent with alterations in hemostasis (see Fig. S1 in the supplemental material).

10.1128/mBio.00473-18.1 FIG S1  Characterization of the host transcriptional response in broad terms by gene set enrichment analyses. The gene sets are as follows: (A) BIOCARTA, (B) KEGG, (C) PID, (D) REACTOME, (E) MOTIF, (F) GO—biological process, (G) GO—cellular components, (H) GO—molecular function, (I) Hallmark. Download FIG S1, TIF file, 0.4 MB. Copyright © 2018 Low et al. 2018 Low et al. This content is distributed under the terms of the Creative Commons Attribution 4.0 International license.

10.1128/mBio.00473-18.5 TABLE S2  Gene set enrichment analyses of upregulated murine genes in infected mice compared to the mock-infected mice. Download TABLE S2, PDF file, 0.4 MB. Copyright © 2018 Low et al. 2018 Low et al. This content is distributed under the terms of the Creative Commons Attribution 4.0 International license.

The most highly induced genes in these categories corresponded to broad functional clustering, especially of genes that encode various cytokines (CSF3, oncostatin M, IL1 family member 9 [IL1F9], IL37, IL6, IL1a, and IL1b) and chemokines (CXCL2, CCL3, CCL4, CXCL1, CCL2, and CCL7) (Table 1). These observations need to be interpreted in the context of an unusual disease where the hallmark of infection is the paucity of a polymorphonuclear leukocyte influx into the lesion, which is potentially a reflection of alpha-toxin-mediated vascular leukostasis in the adjacent blood vessels (4, 9–12). Other studies have shown that PFO can act in synergy with alpha-toxin (5, 12) and can activate expression of cytokines TNF-α and IL6 and subsequent apoptosis (14) as well as expression of IL8 (15) and that injection of PFO into mice activates the production of TNF-α (16, 17), IL1β, IL6, IL10, IL2, and gamma interferon (IFN-γ) (17). Indeed, blocking of TNF-α in mice using neutralizing antibodies caused them to be more resistant to treatment with alpha-toxin, implying a key role for TNF-α in disease pathogenesis (17). Furthermore, treatment with alpha-toxin induced elevated serum levels of CXCL1, a murine homologue of IL8 (18) that is considered to be important in neutrophil recruitment (19). This innate immune response is notable for activation of a skewed NF-κB-regulated proinflammatory response but in the absence of the interferon regulatory factor (IRF)-driven IFN response genes that would typify activation of TLR4 or endosomal TLRs (TLR3, TLR7, TLR8, and TLR9). This result is consistent with activation of TLR2, which is often not associated with an IFN response and is also induced (20). TLR2-mediated NF-κB activation is dependent on the presence of exogenous microbial agents or endogenous inflammatory stimuli from necrotic cells (21), which in mice has been shown to be associated with muscular damage (22–24). Importantly, this activation process is required for *C. perfringens alpha*-toxin-induced reactive oxygen species (ROS) production and cytotoxicity (25).

**TABLE 1:** Selected upregulated host immunity genes^a^

Ensembl ID	Gene name	Product	Log_2_-fold change (WT versus PBS)
ENSMUSG00000058427	*CXCL2*	Growth-regulated alpha protein 2	7.04
ENSMUSG00000038067	*Csf3*	Colony-stimulating factor 3	6.98
ENSMUSG00000000982	*Ccl3*	C-C motif chemokine 3	6.83
ENSMUSG00000032691	*Nlrp3*	Cryopyrin	5.86
ENSMUSG00000025746	*IL6*	Interleukin-6	5.71
ENSMUSG00000018930	*Ccl4*	C-C motif chemokine 4	5.41
ENSMUSG00000042265	*Trem1*	Triggering receptor expressed on myeloid cells 1	5.38
ENSMUSG00000029380	*CXCL1*	Growth-regulated alpha protein 2	5.28
ENSMUSG00000027398	*II1b*	Interleukin-1β	5.14
ENSMUSG00000027399	*II1a*	Interleukin-1α	5.12
ENSMUSG00000029379	*CXCL3*	Growth-regulated alpha protein 3	5.11
ENSMUSG00000078817	*Nlrp12*	Nlrp12 protein	3.65
ENSMUSG00000024401	*Tnf*	Tumor necrosis factor	3.22
ENSMUSG00000027995	*Tlr2*	Toll-like receptor 2	3.06
ENSMUSG00000035385	*Ccl2*	C-C motif chemokine 2	3.03
ENSMUSG00000026180	*Cxcr2*	CXC chemokine receptor	2.39
ENSMUSG00000025225	*NF-κB*	Nuclear factor NF-kappa-B	2.28
ENSMUSG00000035373	*Ccl7*	C-C motif chemokine 7	2.24
ENSMUSG00000022534	*Mefv*	Pyrin	2.23

a Upregulation was defined by an FDR value of <0.01 and a log_2_(fold change) of >2. ID, identifier; WT, wild-type infected mice; PBS, mice treated with phosphate-buffered saline.

A dominant feature of the host genes activated in this response is represented by those associated with inflammasome activation, including the sensor NLRP3 [log_2_(fold change) = 5.86 induction] and IL1β [log_2_(fold change) = 5.14 induction]. NLRP3 mediates both infectious and “sterile” innate immune responses associated with nonpathogenic stimuli such as uric acid and cholesterol crystals (26). It mediates the assembly of an inflammasome signaling complex and a series of proteolytic steps involving cleavage and activation of caspase 1 and pro-IL1 to produce active mature cleaved IL1β (27). Secretion of IL1β may lead to cellular toxicity or pyroptosis, inflammation, and septic shock (28, 29), which are responses that involve some processes that are seen in common with this infection model. However, while there have been other reports of inflammasomes associated with infections (27), the results obtained in this study represent the first indication of NLRP3 involvement in a myonecrosis infection.

It is significant that three CXCL-encoding genes (*CXCL1*, *CXCL2*, and *CXCL3*) and four CCL-encoding genes (*Ccl2*, *Ccl3*, *Ccl4*, and *Ccl7*) were upregulated in the infected mice (Table 1) (Fig. 3), given that infiltration of leucocytes is not a pathological feature of this infection. It may be that chemoattractant signals are sent out from the infected muscle but are not observed due to their being blockaded by platelet-mediated alterations to vasculature and blood clotting. Examples of genes that affect these processes include those encoding thrombospondin 1 [log_2_(fold change) = 3.76] as well as selectins and other adherence factors {endothelial cell selective [log_2_(fold change) = 4.07]}. In addition to being an important mediator of the inflammatory responses that direct the movement of circulating leukocytes to the sites of inflammation (30), CXCL2 also may be involved in muscle fiber regeneration and muscle fiber damage control (31). Recent studies have shown that alpha-toxin adversely affects the innate immune system in a *C. perfringens* infection by inhibiting neutrophil differentiation, although the tissues used in these experiments were taken 24 h after infection (32).

### Global changes in *C. perfringens* gene expression were observed in the infected lesions.

Previous studies have examined the transcriptomes of regulatory mutants of *C. perfringens* JIR325 *in vitro* (33), of an avian necrotic enteritis strain in chicken intestinal loops (34), and of a *C. perfringens* biofilm (35). We compared the *in vivo* bacterial gene expression levels with the expression levels from the equivalent *in vitro* broth cultures, which represents the first time that the transcriptome of *C. perfringens* cells in a myonecrosis infection has been examined. The results showed that host selective pressure induced a global change in *C. perfringens* gene expression. In all, 923 genes, a number which represents approximately 33% of the total genome, were differentially expressed during infection [false-discovery rate (FDR), <0.01; log_2_(fold change), ≥1]. Of these genes, 441 (~48%) were upregulated, whilst 482 (≈52%) were downregulated (Fig. 2A and B) (Table S3).

10.1128/mBio.00473-18.6 TABLE S3  Differentially expressed genes in *C. perfringens* cells grown *in vivo* compared to *in vitro*. Download TABLE S3, PDF file, 0.9 MB. Copyright © 2018 Low et al. 2018 Low et al. This content is distributed under the terms of the Creative Commons Attribution 4.0 International license.

### Genes encoding known or potential virulence factors were differentially regulated in infected lesions.

**FIG 4:**
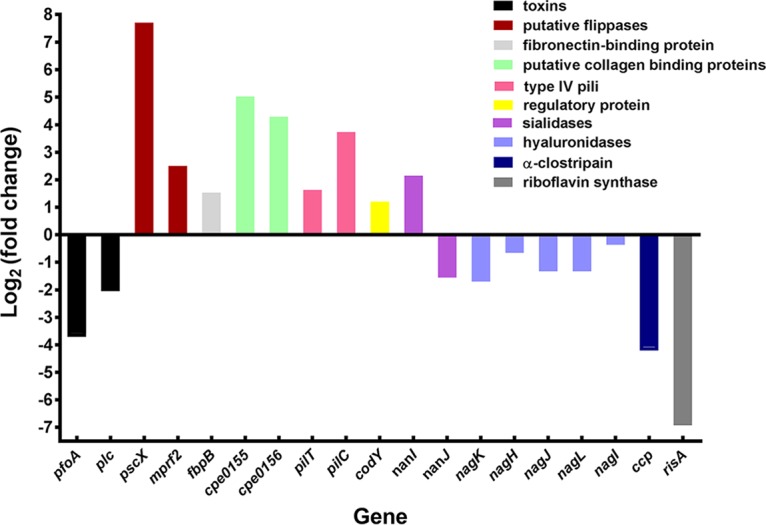
Comparative expression ratios (*in vivo* versus *in vitro*) of genes encoding toxins and potential virulence-associated factors. Refer to Table S3 for details and locus tag data.

The genes that encode the major myonecrosis toxins, i.e., alpha-toxin (*plc* or *cpa*) and PFO (*pfoA*), unexpectedly were expressed at lower levels *in vivo* than *in vitro* [log_2_(fold change) = −1.92 and −3.58, respectively] (Fig. 4). Although the genes were downregulated, there still were 750 reads that mapped to *plc* (compared to 1,583 reads in the *in vitro* samples) and 1,050 reads that aligned to *pfoA* (compared to 7,080 *in vitro* reads), indicating that these toxin genes were expressed *in vivo*. Note that the minimum level of *plc* expression required to cause disease in an infection has not been determined, but it is clear from the histopathology that there was enough alpha-toxin produced after 90 min to initiate the first stages of the observable disease pathology (Fig. 1C). Similarly, genes encoding extracellular hydrolytic enzymes generally were downregulated, whereas genes encoding putative adhesins or proteins that could potentially bind to the host extracellular matrix were upregulated (Fig. 4). The role of most of these genes remains unknown, as does the expression level required for any putative *in vivo* function.

**FIG 5:**
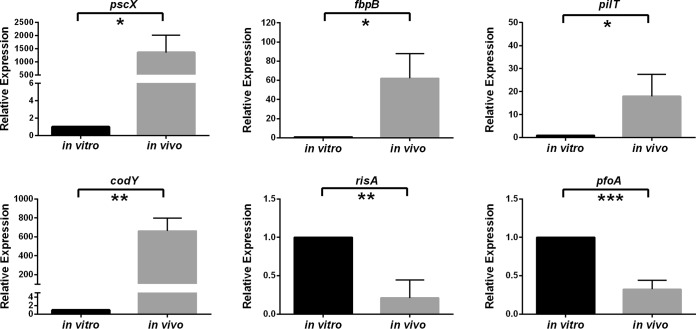
qRT-PCR analysis of selected bacterial genes. Expression levels in cells grown *in vitro* and *in vivo* are shown relative to the respective levels of *rpoA* expression. Values represent means ± standard errors of the means (SEM) of results from three independent biological replicates. Statistically significant differences (*P* ≤ 0.05 by unpaired *t* test) are denoted by asterisks as follows: *, *P* ≤ 0.05; **, *P* ≤ 0.01; ***, *P* ≤ 0.001.

Subsequently, all of the genes that were differentially regulated *in vivo* were examined and those genes that encoded putative virulence factors, or could potentially be involved in the subversion of the host immune response, were selected for further analysis (Fig. 4). To validate the RNA-seq results, six genes that were differentially expressed to different levels were analyzed by qRT-PCR. Four of these genes (*pscX*, *fbpB*, *pilT*, and *codY*) were upregulated *in vivo*, while two (*risA* and *pfoA*) were downregulated *in vivo*. The qRT-PCR results (Fig. 5) correlated with the RNA-seq data, thereby validating the RNA-seq approach.

The *C. perfringens* gene most highly upregulated *in vivo* was *pscX* (CPE0489), which was upregulated by a log_2_(fold change) of 7.7 (Fig. 4; see also Table S3). DELTA-BLAST searches revealed that it had low level similarity to conserved multidrug and toxin extrusion (MATE)-Wzx-like domains (36). Since the PscX protein had 58% identity with a flippase from *Bacillus cereus* (37), we suggest that *pscX* encodes a putative Wzx flippase that translocates glycan subunits. The role of Wzx flippase-mediated membrane translocation in virulence has not been well studied; however, bacterial cell surface glycans mediate many biological functions, such as attachment-colonization, persistence, motility, virulence, and interaction with host defense systems (38, 39). We postulate that PscX may be involved in the synthesis of cell envelope glycans or teichoic acids that have a role in virulence by mediating interactions with host cell surface receptors or extracellular matrix components. We analyzed the host transcriptional response by various GSEA for categories of gene expression changes that might be linked to the induction of this gene in the pathogen (Fig. S1 and Table S2). In the KEGG analysis, the three most significant categories of genes that changed during infection were “ECM receptor interaction,” “cytokine receptor interaction,” and “NLR pathway.” In PID and Reactome analyses, the most significant category of gene changes was “integrins.” Examples of specific genes in these categories include those encoding ECM enzymes such as *Has1* [log_2_(fold change) = 5.18 increase], *Mmp8* [log_2_(fold change) = 4.8], and *ADAMts4* [log_2_(fold change) = 4.69]; those encoding innate immune receptors such as *Nlrp3* [log_2_(fold change) = 5.86], *Clec4e* [log_2_(fold change) = 5.04], and *Clec4d* [log_2_(fold change) = 4.07]; and those encoding cytokine receptors such as CSF3 [log_2_(fold change) = 6.89] and IL1R2 [log_2_(fold change) = 4.21].

The RNA-seq results demonstrated that the c*pe1247* gene, which encodes an orthologue of *mprF2* from *Staphylococcus aureus* (40), was also upregulated *in vivo* [log_2_(fold change) = 2.50] (Fig. 4; see also Table S3). MprF ("multiple peptide resistance factor")-like proteins are conserved membrane proteins that contain synthase and flippase domains. The synthase domain synthesizes l-lysine-phosphatidylglycerol (Lys-PG) or l-alanine-phosphatidylglycerol (Ala-PG), while the flippase domain facilitates the translocation of Lys-PG or Ala-PG to the outer surface of the membrane (41), modifying anionic phospholipids and reducing the negative charge of the cell envelope. This modification increases bacterial resistance to host cationic antimicrobial peptides (CAMPs) such as defensins, cathelicidins, and kinocidins (41, 42) and prokaryotic competitors such as bacteriocins (41). MprF has also been shown to be involved in virulence in pathogens such as *S. aureus*, *Listeria monocytogenes*, and *Mycobacterium tuberculosis* (41). In *M. tuberculosis*, for example, the lysinylation of the membrane by MprF helps the bacterium avoid peptidoglycan degradation, maintaining the optimal membrane potential for bacterial survival upon infection (43).

The putative MprF2 protein from *C. perfringens* strain JIR325 contains both the synthase and flippase domains. The role of MprF in myonecrotic strains of *C. perfringens* such as JIR325 has not been determined. However, recombinant MprF proteins from *C. perfringens* SM101, which is of food poisoning origin, have been shown to aminoacylate peptidoglycan with l-alanine or l-lysine (40). We postulate that MprF2 may play a role in protecting *C. perfringens* cells from host CAMPs during the early stage of infection and therefore may be an important factor for *in vivo* growth and virulence. In terms of a host response to the flippase, potentially in cationic antimicrobial peptide (CAMP) genes, this was not an enriched category in any of the GSEA results (Fig. S1); nor were any individual genes of these categories altered, except for the *Cramp1*-like gene, which was repressed by approximately log_2_(fold change) = 5.

Fibronectin is an ECM glycoprotein that interacts with other ECM molecules, including collagen and fibrinogen. Many pathogenic bacteria, including *C. perfringens*, produce fibronectin-binding proteins (Fbps) that may facilitate their adherence and colonization (44, 45). Two Fbps from *C. perfringens* strain 13, FbpA (CPE0737) and FbpB (CPE1847), have been identified (46), and the recombinant forms of both proteins were reported to bind to the human fibronectin III_1_-C peptide (45). The expression of the *fbpB* gene, but not the *fbpA* gene, was upregulated [log_2_(fold change) of 1.53] *in vivo* (Fig. 4), a result that was validated by qRT-PCR (Fig. 5). It has been shown that recombinant FbpB (rFbpB) has four times more fibronectin-binding activity than rFbpA (47), which may explain why only the expression of *fbpB* was upregulated *in vivo*. These data were consistent with a linked host response, where genes in GSEA categories related to ECM and cell attachment were highly enriched (Fig. S1), with upregulation of individual fibronectin receptor genes such as *itga5* [log_2_(fold change) = 2.49] and *flrt1* [log_2_(fold change) = 2.2].

Two adjacent chromosomal genes that appear to be coordinately expressed, *cpe0155* and *cpe0156*, were significantly upregulated *in vivo* [log_2_(fold change) of 5.02 and 4.30, respectively] (Fig. 4; see also Table S3). Both gene products were predicted to be cell surface proteins by PSORTb analysis and contained a typical CnaB (Cna protein B-type) domain, which is found in *S. aureus* collagen-binding surface proteins SdrC, SrdD, and SdrE (48). In *S. aureus*, this domain forms a stalk in the collagen-binding protein, exposing the functional ligand binding domain on the outer side of the bacterial cell (48, 49). CnaB-like proteins have been shown to be involved in the adherence of *S. aureus* to epithelial cells (50, 51). It has been demonstrated that the CnaB domain is expressed *in vivo* during a *S. aureus* infection and that polyclonal antibodies raised against this domain confer protective immunity by reducing the bacterial load in infected mice (48). It is not known whether CPE0155 or CPE0156 is involved in the adherence of *C. perfringens* to host tissues. However, recent studies have shown that the presence of the CnaA protein, which also has a CnaB domain, correlates with the ability of avian isolates of *C. perfringens* to cause necrotic enteritis in poultry (52). By way of correlation, genes in broad GEO/GSEA categories associated with adherence such as integrins and ECM were highly enriched for changes in expression (Fig. S1). Changes in specific genes in this category include induction of *ADAM8*, *Itga5*, *Itga3*, and *Itga7*.

*C. perfringens*, like many other bacteria, produces type IV pili that extend from the cell envelope and retract into the cell after attaching to an external surface, thereby bringing the bacterium to the site of attachment (53). The *pilT* (*cpe1767*) and *pilC* (*cpe1843*) genes were upregulated *in vivo* [log_2_(fold change) = 1.63 and 3.74, respectively], albeit at low levels (Fig. 4; see also Table S3). PilT belongs to the AAA family of ATPases that oligomerize and hydrolyze ATP for the assembly or disassembly of pili on the cell surface (54), which results in type IV pilus-mediated twitching motility (55). PilC is an integral membrane platform protein that is essential for the biogenesis of type IV pili (56); therefore, *C. perfringens pilC* mutants are unable to produce pili (54). Both PilT and PilC have been shown to be involved in adherence (53) and in gliding or twitching motility and biofilm formation (54) in *C. perfringens*. It also has been shown that *C. perfringens* adheres to mouse and rat myoblasts (muscle cell lines) in a PilT-dependent manner (57). We postulate that these type IV pilus-dependent processes may contribute to the growth and spread of *C. perfringens* in the host.

Since the *C. perfringens* genome lacks many of the genes required for the biosynthesis of amino acids and other metabolites (58, 59), it has been proposed that *C. perfringens* produces many hydrolytic extracellular enzymes that degrade extracellular host components to obtain nutrients from the host for its growth and survival (60). However, the RNA-seq results showed that genes that encode putative hydrolytic enzymes such as hyaluronidases (NagK, NagH, NagJ, NagL, and NagI), the cysteine protease α-clostripain (Ccp), collagenase (ColA), and sialidase (NanJ) were downregulated *in vivo*. In contrast, the gene that encodes the major sialidase, NanI, was upregulated *in vivo* (Fig. 4). Accordingly, there was a significant induction of expression of host genes associated with degradation of the ECM and the generation of metabolites (Fig. S1).

Sialidases hydrolyze terminal sialic acid linkages in various surface sialoglycoproteins, which subsequently may be transported into bacterial cells as a nutrient source (61). They have also been shown to contribute to virulence in many bacteria and have been implicated in the pathogenesis of myonecrosis infections, with evidence indicating that the removal of sialic acids by *C. perfringens* NanI increases the sensitivity of host cells to alpha-toxin (62, 63). The upregulation of *nanI* observed here suggests that NanI may be important for bacterial growth and virulence during early infection. Previous studies have shown that neither NanI nor NanJ is essential for virulence in the murine myonecrosis model; however, this model would not have detected effects that are specific to the early stages of infection (64). There are many extensively glycosylated cell surface receptors (Trem1, Clec receptors, and IL1R as well as secreted glycoproteins) that are induced in response to infection and that may be the source of substrates for the NanI sialidase.

Finally, more than 20 known or putative regulatory genes, including *revR*, *virR*, *reeS*, *codY*, *tex*, and *ccpA*, were up- or downregulated *in vivo* (Table S4). These results suggest that there are previously uncharacterized transcriptional regulators that may have a role in the early stages of infection. In addition, we recently identified 93 potential small RNA (sRNA) molecules in *C. perfringens* (33), many of which may have a regulatory role. In this study, we have shown that 16 of these sRNA genes were upregulated *in vivo* and 9 were downregulated (Table S3). Correspondingly, there also were host cell regulatory genes (e.g., *ATF2*, *AP-1*, *GR*, and *NFAT*) whose expression was altered in the infected lesions, as indicated in the PID GSEA categories (Fig. S1). Specific genes induced include those encoding the transcription factors Fos [log_2_(fold change) = 4.27], JunB [log_2_(fold change) = 4.26], and Nfkbiz [log_2_(fold change) = 3.97]. In addition, there are numerous regulatory genes that were significantly downregulated, for example, small nucleolar RNA 23 (snoRNA23), snoRNA69, and snoRNA118 and genes encoding 10 zinc finger proteins that were among the 20 most highly downregulated genes.

10.1128/mBio.00473-18.7 TABLE S4  Differentially expressed regulatory genes. Download TABLE S4, PDF file, 0.3 MB. Copyright © 2018 Low et al. 2018 Low et al. This content is distributed under the terms of the Creative Commons Attribution 4.0 International license.

### The expression of plasmid-borne genes was altered *in vivo*.

*C. perfringens* strain 13 and its derivatives carry a *cpb2* toxin-encoding plasmid, pCP13 (58). pCP13 carries 63 genes, some of which are expressed at very low levels, which means that designations of up- or downregulated genes need to be interpreted conservatively. Among the pCP13-carried genes, 28 were differentially expressed *in vivo*, 15 at a lower level and 13 at a higher level (Table S5). Presumptive upregulated genes encoded a putative MerR family regulatory protein (PCP12), transposases (PCP05 and PCP10), a resolvase (PCP32), and PemK (PCP58), which is an orthologue of a toxic RNase that is part of a toxin-antitoxin module involved in stable plasmid maintenance in other bacteria (65, 66). Plasmid-encoded toxin-antitoxin systems are often activated in response to environmental stress, which is consistent with our observation that the *pemK* orthologue on pCP13 was upregulated *in vivo*. Previous studies have shown that the expression of some pCP13 genes is regulated by the VirSR-VR-RNA regulatory network (33, 67) and, to a lesser extent, by RevR (33). However, the data presented here represent the first evidence that pCP13-carried genes are differentially regulated *in vivo*.

10.1128/mBio.00473-18.8 TABLE S5  Differentially expressed pCP13 genes. Download TABLE S5, PDF file, 0.3 MB. Copyright © 2018 Low et al. 2018 Low et al. This content is distributed under the terms of the Creative Commons Attribution 4.0 International license.

### *C. perfringens* modifies the expression of its metabolic genes under *in vivo* growth conditions.

To identify functions and pathways whose expression in *C. perfringens* was enriched during an infection, the upregulated genes were categorized according to their GO and KEGG functions. Major metabolic genes that were significantly upregulated *in vivo* [FDR, <0.01; log_2_(fold change) > 1] included clusters of genes involved in the purine and pyrimidine biosynthesis pathways, as well as genes encoding ABC transporters and involved in putative peptide and metal ion transport systems (Fig. S2; see also Table S3), all of which was consistent with the growth requirements of *C. perfringens* in an infected lesion. Expression levels of host response genes involved in purine metabolism, including the innate immune associated purinergic receptors (P2RX7, P2RY), either were not dramatically changed or were decreased only slightly.

10.1128/mBio.00473-18.2 FIG S2  KEGG pathway and gene ontology (GO) enrichment analysis of significantly upregulated *C. perfringens* genes during *in vivo* growth. Circles denote enriched KEGG categories, while triangles denote enriched GO categories. Percentages of enriched genes represent the proportions of genes in that category that are differentially expressed. BR, BRITE, a computer representation of molecular systems [M. Kanehisa, S. Goto, Y. Sato, M. Furumichi, and M. Tanabe, 2012, Nucleic Acids Res. **40**(Database issue)**:**D109–D114]. Download FIG S2, TIF file, 0.7 MB. Copyright © 2018 Low et al. 2018 Low et al. This content is distributed under the terms of the Creative Commons Attribution 4.0 International license.

Iron is an essential element for the growth of all living cells; consequently, the limited supply of iron in the host represents a defense mechanism against bacterial infections (68). To overcome this host iron limitation, pathogenic bacteria have evolved numerous iron uptake mechanisms (68, 69). The RNA-seq results showed that expression of several genes that potentially encode components involved in iron uptake was upregulated *in vivo*. These genes included *feoAB* (cpe*1660*) and the *chtE srt chtA* (*cpe0221* to *cpe0223*) gene region. We have recently shown that *feoB* encodes the major ferrous iron uptake protein in *C. perfringens* strain 13 (70). ChtE is a heme binding protein that is located in the cell envelope, ChtA is a putative periplasmic binding protein that is predicted to be part of a heme-specific ABC transport system, and the product of the *srt* gene is a putative sortase protein (71). Again, the upregulation of these genes is consistent with the *in vivo* growth requirements of *C. perfringens* cells. With regard to the host response, there do not appear to be any dramatic changes in host genes encoding components of iron metabolism (heme biosynthesis and carrier proteins such as transferring and heme-containing enzymes of the cytochrome family).

### Conclusions.

In this study, concurrent RNA-seq analyses of both *C. perfringens* and the host in a murine myonecrosis infection were used to obtain a comprehensive view of both host and pathogen gene expression in infected muscle tissues. The results showed that there were major changes in the *C. perfringens* transcriptional profile in the host environment. *C. perfringens* genes that may be involved in replication, virulence, subversion of host immune systems, and adaptation of bacterial metabolism to host conditions were upregulated. The concurrent host gene expression profile was characterized by the activation of genes that were involved in innate immunity, including the NLRP3 inflammasome pathway, and in changes that responded to the observed alteration in the production of bacterial gene products. These results represent the first transcriptional profiles of the host and pathogen in a histotoxic bacterial infection. The results have opened the way for more-detailed studies on the mechanism of host-pathogen interactions in a *C. perfringens* infection.

## MATERIALS AND METHODS

### Bacterial strains and growth conditions.

The bacterial strain used in this study was *C. perfringens* strain JIR325, a rifampin-resistant and nalidixic acid-resistant derivative of strain 13 (72). It was cultured at 37°C in tryptone-peptone-glucose (TPG) broth, fluid thioglycolate (FTG) medium (Difco), or nutrient agar supplemented with 10 μg/ml rifampin (RIF) and 10 μg/ml nalidixic acid. Culture media and antibiotics were from Oxoid and Sigma, respectively, unless otherwise stated. All agar cultures of *C. perfringens* were incubated under anaerobic conditions (10% [vol/vol] H_2_, 10% [vol/vol] CO_2_, and 80% [vol/vol] N_2_) at 37°C.

### Virulence studies.

Myonecrosis infections were performed using 6-to-8-week-old female BALB/c mice as previously described (73, 74), with some modifications. First, both hind legs of the mice were injected intramuscularly with 50 μl of washed cells in sterile Dulbecco’s phosphate-buffered saline (PBS), which is equivalent to approximately 10^9^ CFU. Second, mice were euthanized 1.5 h after infection, except for one mouse in each experiment, which was used to monitor overall disease progression. The infected hind muscles were dissected from the euthanized mice and were immediately placed in microcentrifuge tubes containing 1 ml of RNAlater (Ambion) and incubated on ice or stored at −80°C. Mice in the mock-infected group were injected with 50 μl of PBS only. All animal experiments were conducted in accordance with Victorian State Government regulations and were approved by the Monash University SOBS B Animal Ethics Committee.

### Isolation of total RNA from the *C. perfringens-*infected and mock-infected mice muscle lesions.

The infected muscle tissue stored at −80°C in RNAlater (Ambion) was thawed on ice, transferred into a tube that contained 1 ml of TRIzol (Invitrogen), and homogenized with a Precellys 24 homogenizer (Bertin Technologies) using ≤106-µm-diameter sterile acid-washed glass beads (Sigma) that had been washed three times with PBS. Homogenization released *C. perfringens* cells from the infected muscle tissue and facilitated bacterial and murine RNA isolation. The homogenate was filtered through a cell strainer and then centrifuged for 8 min at 16,060 × *g* at 4°C to recover the tissue and bacterial cells from the RNAlater. The pellet, which comprised both host tissues and bacterial cells, was resuspended in ice-cold 0.2 M sucrose–0.01% (wt/vol) SDS (75). Bacterial and murine total RNA extraction was performed as previously described (76, 77). For the infected mice, RNA samples isolated from four mice (eight thighs) were pooled to form one biological replicate. For the mock-infected mice, RNA samples isolated from two mice (four thighs) were pooled to form one biological replicate. Pooled RNA samples were prepared in triplicate.

Each pooled RNA sample was divided into two aliquots for host and pathogen RNA-seq reactions. One fraction was subjected to bacterial mRNA enrichment by subtracting eukaryotic poly(A)-mRNA and bacterial rRNA from the sample; the other aliquot was used for host RNA-seq analysis. Totals of at least 4.5 million and 147 million reads were obtained from the pathogen and host RNA-seq experiments, respectively (see Table S6 in the supplemental material). Since bacterial RNA depletion was not carried out on the RNA used for host RNA-seq, approximately 12% of the total reads mapped to the *C. perfringens* genome; these reads were excluded from the host data analysis.

10.1128/mBio.00473-18.9 TABLE S6  Summary of mapped *C. perfringens* and murine RNA-seq reads. Download TABLE S6, PDF file, 0.1 MB. Copyright © 2018 Low et al. 2018 Low et al. This content is distributed under the terms of the Creative Commons Attribution 4.0 International license.

### Isolation of total RNA from *in vitro C. perfringens* cultures.

Cells were grown at 37°C overnight on nutrient agar supplemented with 10 μg/ml RIF and 10 μg/ml nalidixic acid. The cells were collected from the plates and washed three times with sterile PBS. Bacterial cells were then pelleted, and the volume of cell pellet was recorded. The pellet was then suspended in 2 volumes of PBS and the viable count determined. Subsequently, 250 μl of the diluted washed cells, which was equivalent to approximately 5 × 10^9^ CFU, was added to 5 ml of TPG broth, with the intention of mimicking the *in vivo* inoculum. After 90 min at 37°C, the cells were harvested by centrifugation at 8,200 × *g* for 10 min at room temperature. Total RNA was then isolated using TRIzol reagent (Invitrogen) as previously described (33, 76, 77).

### Determination of RNA quality.

The integrity and quantity of the final enriched RNA preparations were determined using a 2100 Bioanalyzer microfluidic system (Agilent Technologies), unless otherwise stated. The results from microfluidic gel electrophoresis were visualized in digital electropherograms, and simulated gel views were generated using 2100 Expert software (Agilent Technologies). The software scored RNA sample integrity on a scale of 10, where an RNA integrity number (RIN) of 10 represented the highest quality of intact RNA with minimal degradation and a RIN of 1 marked a completely degraded RNA sample. To ensure high-quality RNA-seq reads, and to ensure that the host and pathogen RNA peaks could be distinguished from one another, only RNA samples with a RIN of greater than 6.8 were used in this study.

### Bacterial mRNA enrichment and rRNA depletion.

The RNA aliquot was treated with Turbo DNase (Ambion) at 37°C for 30 to 60 min to eliminate DNA contamination. The resultant RNA was then used as a template for the PCR amplification of a housekeeping gene, *rpoA* (see Table S7 for primers); a negative PCR result provided evidence that the RNA was free of DNA contamination. PCR cycling conditions were as follows: 95°C for 5 min, followed by 30 cycles of 95°C for 1 min, 55°C for 1 min, and 72°C for 1 min. Subsequently, the contaminating host RNA was depleted from the sample using a MICROBEnrich kit (Ambion) in accordance with the manufacturer’s instructions. A total of 5 μg of pooled RNA was subjected to repeated cycles of bacterial RNA enrichment to ensure the almost complete depletion of the host RNA. Next, bacterial rRNA was depleted from the preparation using a Ribo-Zero rRNA removal kit (Epicentre) per the manufacturer’s instructions. The quality and quantity of RNA obtained after the bacterial RNA enrichment and rRNA depletion were determined using a 2100 Bioanalyzer.

10.1128/mBio.00473-18.10 TABLE S7  Oligonucleotide primers for qRT-PCRs. Download TABLE S7, PDF file, 0.3 MB. Copyright © 2018 Low et al. 2018 Low et al. This content is distributed under the terms of the Creative Commons Attribution 4.0 International license.

### Bacterial library preparation.

Libraries were prepared for use in bacterial RNA-seq analysis using Illumina TruSeq RNA sample preparation kit v2, following the manufacturer’s instructions with a few modifications. The first step of the library preparation protocol, which involves purifying poly(A) mRNA molecules from total RNA, was omitted, and 400 ng of bacterial mRNA was used in each library preparation. RNA-seq analysis of the enriched RNA samples from the *in vivo*- and *in vitro-*derived cells was carried out on Illumina HiSeq and MiSeq instruments, respectively. Analysis of the heat maps (see Fig. S3 in the supplemental material) generated from the RNA-seq data showed that gene expression profiles in the three biological replicates in each experimental group clustered together and had similar patterns, providing evidence that there was a high level of reproducibility among the replicates in each group.

10.1128/mBio.00473-18.3 FIG S3  Heat maps derived from host and pathogen RNA-seq. Data were analyzed with Voom-limma (83). Each row represents expression data from one gene normalized to the row mean. Only genes that were significantly upregulated (red) or downregulated (blue) [FDR of <0.01 and log_2_ (fold change) of >1] in the murine host or *C. perfringens* are shown. I, mice infected with JIR325; M, mice mock-infected with PBS; B, broth culture. The biological replicates are designated 1, 2, and 3. Download FIG S3, TIF file, 0.3 MB. Copyright © 2018 Low et al. 2018 Low et al. This content is distributed under the terms of the Creative Commons Attribution 4.0 International license.

### Host RNA-seq.

The second aliquot of total RNA extracted from infected mouse thigh muscle tissue was treated with Turbo DNase (Ambion) at 37°C for 30 to 60 min and then purified using an RNeasy column (Qiagen). RNA integrity was then measured as described above. A total of 5 μg of this RNA was depleted of both eukaryotic and bacterial rRNA, using an 8:2 ratio of eukaryote/bacterium probe mix. The rRNA-depleted samples then were analyzed using an Agilent Bioanalyzer to determine the level of rRNA contamination. RNA samples with greater than 8% rRNA contamination were not analyzed further. Subsequently, 300 ng of rRNA-depleted RNA was enzymatically sheared with RNase III (Life Technologies, Inc.) at 37°C for 10 min. Where possible, 100 ng of sheared RNA was used for each library preparation using a SOLiD total RNA-seq kit (Life Technologies, Inc.). Each library concentration was determined using the HighSense Agilent bioanalyzer assay per the instructions of the manufacturer (Agilent Technologies). Libraries were sequenced using a SOLiD 5500xl genetic analyzer.

### Analysis of RNA-seq data.

Bacterial sequenced reads were aligned to the *C. perfringens* strain 13 reference genome sequence (GenBank accession numbers NC_003366 and NC_003042) using SHRiMP alignment software (78). The number of reads aligning to each genome location, including the open reading frames and intergenic regions, was determined using Nesoni (https://github.com/Victorian-Bioinformatics-Consortium/nesoni). The coverage plot for each sample was then generated by Nesoni and was visualized in the Artemis genome browser (79) or Integrative Genome Viewer (80, 81). RPM (reads assigned per million mapped reads) normalization of the RNA-seq data was employed in this study as follows: the total number of reads mapped to a gene was divided by the total number of million mapped reads in each sample [mapped reads per gene (location)/million reads mapped to genome] (82). The data have been lodged in the GEO repository (see below).

Murine sequenced reads were mapped against the *Mus musculus* GRCm38 genome (mm9) using Thermo (Fisher) Lifescope software, version 2.5.1. The *C. perfringens* genome was included as an artificial chromosome to filter out any DNA of bacterial origin that had been included among the murine reads. Data normalization was again performed by expressing reads per gene as a fraction of total mapped reads. The data have been lodged in the GEO repository (see below).

Genes that were differentially expressed between the *in vivo* and *in vitro* bacterial samples, or between the infected mice and the PBS mock-infected mice, were identified using the voom-limma analysis package (83) with a false-discovery rate (FDR) value of 0.01 and an up- or downregulated log_2_(fold change) value of >1. The transcriptional response was classified in broad terms by GO (gene ontology) and GSEA (gene set enrichment analyses), and we have used several of these analyses to obtain broad categories by which to describe the nature of the host response. These often have different emphases such as broad biological processes (KEGG), protein interactions (PID), or molecular pathways (biocarta) or combinations (Hallmark). Functional characterization categories for upregulated bacterial and host genes were also identified using the bioconductor “GOseq” package (84) and analysis tools provided by the DAVID (database for annotation, visualization and integrated discovery) online database (85).

### Quantitative reverse transcription (qRT) analysis.

To validate the RNA-seq results, qRT-PCR was performed on selected bacterial and host genes. For the bacterial genes, cDNA was synthesized from 8 ng of bacterial mRNA, using 1 μl (10 U/μl) of avian myeloblastosis virus (AMV) reverse transcriptase, 1 μl of 40 mM deoxynucleotide triphosphates, 1 μl (20 to 40 U/μl) of RNasin RNase inhibitor, and 1 μl of random hexamers. All reagents and enzymes were supplied by Promega unless otherwise stated. No-reverse-transcriptase (NRT) control samples were prepared by replacing AMV reverse transcriptase with nuclease-free water. The reaction mixtures were then incubated at 42°C for 1 h, followed by a 90°C incubation for 5 min and, last, a 5-min incubation on ice. For each sample, a 25-μl qRT reaction mixture that contained 12.5 μl SYBR green Master Mix (Applied Biosystems), 1 μl of a 10 μM concentration of each gene-specific forward and reverse primer (Table S7), 1 μl of cDNA, and the appropriate amount of nuclease-free water was prepared. All qRT-PCR reactions were performed using a Mastercycler ep *Realplex* qRT-PCR system (Eppendorf) as previously described (73), except that the initial denaturation time applied in this study was 15 min and the annealing temperature was 50°C. To validate the expression results determined for the selected murine host genes, qRT-PCR was performed in a similar manner using SYBR chemistry (Life Technologies, Inc.) and an automated 7900HT qRT-PCR PCR system (Life Technologies, Inc.). All results were analyzed using the threshold cycle (ΔΔ*C*_*T*_) method (86) and are presented as the expression level of each target gene relative to that of the *rpoA* housekeeping gene (for the bacterial genes) and the 18S gene (for the murine genes).

### Accession number(s).

RNA-seq data determined in this work have been lodged in the GEO repository (GenBank accession numbers GSE96890 and GSE106657).
