# Supplementary material for: Concurrent Host-Pathogen Transcriptional Responses in a Clostridium perfringens Murine Myonecrosis Infection
Source: mBio. 2018 Mar 27;9(2):e00473-18. doi: 10.1128/mBio.00473-18 (PMC5874911; doi:10.1128/mBio.00473-18)
Supplement: TABLE S1 [file mbo002183811st1.pdf]

TABLE S1 Differentially expressed murine genes in muscle tissues from infected compared to the mock-infected mice*.

Ensembl ID	Gene[a]	Log_2 Fold Change	FDR
ENSMUSG00000058427	*Cxcl2*	7.04	9.13E-08
ENSMUSG00000038067	*Csf3*	6.98	3.28E-05
ENSMUSG00000000982	*Ccl3*	6.83	2.90E-05
ENSMUSG00000058755	*Osm*	5.88	5.17E-06
ENSMUSG00000032691	*Nlrp3*	5.86	1.28E-07
ENSMUSG00000044103	*Il1f9*	5.78	1.08E-05
ENSMUSG00000025746	*Il6*	5.71	3.60E-08
ENSMUSG00000086849	*Gm13840*	5.59	4.58E-04
ENSMUSG00000022126	*Irg1*	5.50	7.79E-07
ENSMUSG00000018930	*Ccl4*	5.41	1.93E-05
ENSMUSG00000042265	*Trem1*	5.38	4.04E-06
ENSMUSG00000029380	*Cxcl1*	5.28	3.47E-08
ENSMUSG00000003665	*Has1*	5.18	6.88E-07
ENSMUSG00000091387	*Gcnt4*	5.15	3.02E-05
ENSMUSG00000027398	*Il1b*	5.14	9.13E-08
ENSMUSG00000027399	*Il1a*	5.12	4.29E-06
ENSMUSG00000029379	*Cxcl3*	5.11	5.81E-04
ENSMUSG00000040026	*Saa3*	5.07	7.71E-03
ENSMUSG00000030142	*Clec4e*	5.04	6.03E-08
ENSMUSG00000032487	*Ptgs2*	4.96	1.34E-09
ENSMUSG00000005800	*Mmp8*	4.80	5.69E-06
ENSMUSG00000035963	*Odf3l2*	4.70	3.97E-05
ENSMUSG00000054855	*Rnd1*	4.70	1.14E-05
ENSMUSG00000006403	*Adamts4*	4.69	3.86E-06
ENSMUSG00000026582	*Sele*	4.67	2.88E-06
ENSMUSG00000000182	*Fgf23*	4.64	3.68E-03
ENSMUSG00000046223	*Plaur*	4.55	1.82E-07
ENSMUSG00000022651	*Retnlg*	4.47	1.95E-05
ENSMUSG00000045551	*Fpr1*	4.39	3.29E-04
ENSMUSG00000038418	*Egr1*	4.35	2.17E-08
ENSMUSG00000079597	*Gm5483*	4.32	4.75E-03
ENSMUSG00000030474	*Siglece*	4.29	5.12E-03
ENSMUSG00000003545	*Fosb*	4.27	1.07E-04
ENSMUSG00000052837	*Junb*	4.26	1.97E-09
ENSMUSG00000090698	*Apold1*	4.25	2.70E-09
ENSMUSG00000026073	*Il1r2*	4.21	7.85E-06
ENSMUSG00000026580	*Selp*	4.15	6.88E-07
ENSMUSG00000087684	*1200007C13Rik*	4.15	1.03E-03
ENSMUSG00000026981	*Il1rn*	4.11	1.78E-07
ENSMUSG00000048572	*E030010A14Rik*	4.10	1.50E-05
ENSMUSG00000030144	*Clec4d*	4.07	2.32E-06

ENSMUSG00000027360	*Hdc*	4.01	5.29E-08
ENSMUSG00000041754	*Trem3*	4.01	2.15E-03
ENSMUSG00000035356	*Nfkbiz*	3.97	1.34E-09
ENSMUSG00000032515	*Csrnp1*	3.95	5.68E-09
ENSMUSG00000053560	*Ier2*	3.95	4.04E-07
ENSMUSG00000009292	*Trpm2*	3.90	2.12E-05
ENSMUSG00000052212	*Cd177*	3.84	3.63E-04
ENSMUSG00000020205	*Phlda1*	3.82	5.67E-05
ENSMUSG00000036067	*Slc2a6*	3.81	5.71E-05
ENSMUSG00000056071	*S100a9*	3.81	3.52E-08
ENSMUSG00000040152	*Thbs1*	3.79	1.34E-09
ENSMUSG00000044162	*Tnip3*	3.79	4.04E-06
ENSMUSG00000019850	*Tnfaip3*	3.75	1.56E-09
ENSMUSG00000037405	*Icam1*	3.73	1.80E-08
ENSMUSG00000026822	*Lcn2*	3.73	2.26E-07
ENSMUSG00000021250	*Fos*	3.73	5.08E-09
ENSMUSG00000047798	*Cd300lf*	3.72	8.06E-06
ENSMUSG00000021553	*Slc28a3*	3.71	7.58E-03
ENSMUSG00000089672	*Gp49a*	3.66	1.63E-07
ENSMUSG00000078817	*Nlrp12*	3.65	6.18E-03
ENSMUSG00000036040	*Adamtsl2*	3.64	1.94E-06
ENSMUSG00000037411	*Serpine1*	3.61	1.34E-09
ENSMUSG00000086564	*Cd101*	3.61	1.10E-03
ENSMUSG00000061878	*Sphk1*	3.60	2.26E-06
ENSMUSG00000051439	*Cd14*	3.58	1.95E-08
ENSMUSG00000087242	*C78197*	3.58	4.91E-03
ENSMUSG00000056054	*S100a8*	3.55	1.63E-07
ENSMUSG00000044786	*Zfp36*	3.54	1.58E-09
ENSMUSG00000038729	*Akap2*	3.53	3.85E-03
ENSMUSG00000021123	*Rdh12*	3.53	5.01E-06
ENSMUSG00000024737	*Slc15a3*	3.53	1.51E-05
ENSMUSG00000013974	*1810033B17Rik*	3.49	2.39E-04
ENSMUSG00000020108	*Ddit4*	3.48	2.26E-07
ENSMUSG00000031303	*Map3k15*	3.47	4.58E-03
ENSMUSG00000016529	*Il10*	3.47	7.51E-03
ENSMUSG00000042622	*Maff*	3.46	3.52E-08
ENSMUSG00000048489	*8430408G22Rik*	3.45	9.04E-07
ENSMUSG00000000204	*Slfn4*	3.40	3.30E-06
ENSMUSG00000028967	*Errfi1*	3.38	1.43E-08
ENSMUSG00000020423	*Btg2*	3.36	1.09E-08
ENSMUSG00000066170	*E230001N04Rik*	3.34	3.65E-03
ENSMUSG00000023903	*Mmp25*	3.33	4.68E-03
ENSMUSG00000003752	*Itpkc*	3.32	1.43E-08
ENSMUSG00000048003	*Catsper4*	3.31	7.37E-03

ENSMUSG00000054204	*Fam150b*	3.29	9.16E-03
ENSMUSG00000053113	*Socs3*	3.28	5.74E-07
ENSMUSG00000087006	*Gm13889*	3.28	3.82E-03
ENSMUSG00000003484	*Cyp4f18*	3.27	3.13E-06
ENSMUSG00000075122	*Cd80*	3.26	5.61E-06
ENSMUSG00000022602	*Arc*	3.26	5.70E-05
ENSMUSG00000027737	*Slc7a11*	3.25	1.94E-06
ENSMUSG00000026475	*Rgs16*	3.25	1.47E-04
ENSMUSG00000018476	*Kdm6b*	3.24	9.11E-08
ENSMUSG00000021125	*Arg2*	3.24	2.82E-06
ENSMUSG00000024401	*Tnf*	3.22	2.83E-05
ENSMUSG00000048621	*Gm6377*	3.21	3.94E-05
ENSMUSG00000024190	*Dusp1*	3.20	9.71E-08
ENSMUSG00000024912	*Fosl1*	3.19	5.10E-03
ENSMUSG00000084956	*Gm16194*	3.16	5.26E-03
ENSMUSG00000044313	*Mab21l3*	3.16	1.98E-03
ENSMUSG00000034394	*Lif*	3.15	1.64E-04
ENSMUSG00000034765	*Dusp5*	3.12	1.79E-06
ENSMUSG00000027832	*Ptx3*	3.11	1.21E-07
ENSMUSG00000043953	*Ccrl2*	3.11	4.62E-07
ENSMUSG00000074788	*5830416P10Rik*	3.10	3.26E-04
ENSMUSG00000030187	*Klra2*	3.09	2.52E-03
ENSMUSG00000025473	*Adam8*	3.08	9.13E-08
ENSMUSG00000059657	*Stfa2l1*	3.08	7.99E-03
ENSMUSG00000002578	*Ikzf4*	3.08	6.12E-03
ENSMUSG00000036931	*Nfkbid*	3.07	4.67E-04
ENSMUSG00000027995	*Tlr2*	3.06	3.47E-08
ENSMUSG00000064147	*Rab44*	3.05	1.37E-04
ENSMUSG00000017737	*Mmp9*	3.05	1.23E-04
ENSMUSG00000031980	*Agt*	3.04	2.68E-06
ENSMUSG00000035385	*Ccl2*	3.03	2.59E-06
ENSMUSG00000031762	*Mt2*	3.01	1.82E-07
ENSMUSG00000031488	*Rab11fip1*	3.01	3.74E-05
ENSMUSG00000024042	*Sik1*	3.01	7.94E-09
ENSMUSG00000028195	*Cyr61*	3.00	5.73E-09
ENSMUSG00000026121	*Sema4c*	2.99	1.24E-07
ENSMUSG00000071637	*Cebpd*	2.97	1.19E-07
ENSMUSG00000028862	*Map3k6*	2.97	1.60E-07
ENSMUSG00000053475	*Tnfaip6*	2.93	8.90E-07
ENSMUSG00000015312	*Gadd45b*	2.92	6.44E-07
ENSMUSG00000079243	*Xirp1*	2.91	4.04E-07
ENSMUSG00000041449	*Serpina3h*	2.91	3.40E-03
ENSMUSG00000024427	*Spry4*	2.91	3.96E-07
ENSMUSG00000045502	*Niacr1*	2.90	1.48E-05

ENSMUSG00000021025	*Nfkbia*	2.90	2.20E-08
ENSMUSG00000052749	*Trim30b*	2.88	1.06E-03
ENSMUSG00000028214	*Gem*	2.87	5.84E-07
ENSMUSG00000029371	*Cxcl5*	2.86	9.07E-04
ENSMUSG00000028859	*Csf3r*	2.86	3.71E-05
ENSMUSG00000064246	*Chi3l1*	2.85	5.20E-03
ENSMUSG00000055994	*Nod2*	2.85	1.29E-05
ENSMUSG00000028680	*Plk3*	2.85	1.32E-05
ENSMUSG00000040435	*Ppp1r15a*	2.85	2.35E-07
ENSMUSG00000049130	*C5ar1*	2.84	3.57E-05
ENSMUSG00000026819	*Slc25a25*	2.80	8.40E-06
ENSMUSG00000039232	*Stx11*	2.79	8.02E-06
ENSMUSG00000023034	*Nr4a1*	2.79	3.67E-08
ENSMUSG00000053175	*Bcl3*	2.78	1.75E-04
ENSMUSG00000079293	*Clec7a*	2.78	1.07E-04
ENSMUSG00000037868	*Egr2*	2.78	4.74E-06
ENSMUSG00000040264	*Gbp5*	2.75	4.05E-06
ENSMUSG00000062593	*Lilrb4*	2.74	1.67E-07
ENSMUSG00000029321	*Slc10a6*	2.74	9.24E-06
ENSMUSG00000023067	*Cdkn1a*	2.74	2.26E-07
ENSMUSG00000033508	*Asprv1*	2.73	3.27E-03
ENSMUSG00000068699	*Flnc*	2.72	1.24E-07
ENSMUSG00000001156	*Mxd1*	2.71	1.58E-07
ENSMUSG00000017009	*Sdc4*	2.70	8.68E-09
ENSMUSG00000047945	*Marcksl1*	2.70	3.02E-05
ENSMUSG00000047443	*Fam132b*	2.68	1.93E-03
ENSMUSG00000026840	*Lamc3*	2.67	7.57E-04
ENSMUSG00000054364	*Rhob*	2.66	3.87E-08
ENSMUSG00000080810	*Gm11737*	2.66	4.16E-03
ENSMUSG00000024014	*Pim1*	2.64	1.07E-06
ENSMUSG00000006445	*Epha2*	2.63	6.98E-06
ENSMUSG00000020120	*Plek*	2.61	2.20E-08
ENSMUSG00000031504	*Rab20*	2.61	7.48E-05
ENSMUSG00000040907	*Atp1a3*	2.60	7.15E-03
ENSMUSG00000029135	*Fosl2*	2.60	1.45E-08
ENSMUSG00000052270	*Fpr2*	2.58	3.40E-03
ENSMUSG00000018899	*Irf1*	2.57	9.13E-08
ENSMUSG00000034936	*Arl4d*	2.57	2.63E-06
ENSMUSG00000037447	*Arid5a*	2.55	3.09E-07
ENSMUSG00000056501	*Cebpb*	2.55	6.03E-08
ENSMUSG00000035640	*Dos*	2.55	3.38E-03
ENSMUSG00000038587	*Akap12*	2.52	2.75E-08
ENSMUSG00000050370	*Ch25h*	2.51	1.97E-03
ENSMUSG00000003541	*Ier3*	2.51	6.06E-06

ENSMUSG00000022528	*Hes1*	2.50	1.86E-06
ENSMUSG00000032501	*Trib1*	2.49	2.75E-08
ENSMUSG00000000555	*Itga5*	2.49	3.52E-08
ENSMUSG00000044006	*Cilp2*	2.47	1.46E-05
ENSMUSG00000009772	*Nuak2*	2.46	1.48E-05
ENSMUSG00000003153	*Slc2a3*	2.45	5.55E-06
ENSMUSG00000027068	*Dhrs9*	2.45	2.33E-05
ENSMUSG00000043740	*B430306N03Rik*	2.44	2.08E-03
ENSMUSG00000035621	*Midn*	2.43	1.86E-07
ENSMUSG00000038894	*Irs2*	2.43	4.43E-07
ENSMUSG00000004609	*Cd33*	2.43	4.09E-06
ENSMUSG00000078816	*Prkcc*	2.41	3.25E-03
ENSMUSG00000030022	*Adamts9*	2.39	3.60E-08
ENSMUSG00000026180	*Cxcr2*	2.39	1.36E-03
ENSMUSG00000079254	*Itprip*	2.39	1.19E-05
ENSMUSG00000028834	*Trim63*	2.38	1.83E-07
ENSMUSG00000074001	*Kbtbd5*	2.38	8.70E-07
ENSMUSG00000045534	*Kcna5*	2.38	4.05E-06
ENSMUSG00000024486	*Hbegf*	2.38	2.35E-07
ENSMUSG00000039911	*Spsb1*	2.37	9.03E-06
ENSMUSG00000026459	*Myog*	2.36	5.01E-06
ENSMUSG00000042677	*Zc3h12a*	2.34	6.81E-07
ENSMUSG00000035671	*Zswim4*	2.34	3.75E-06
ENSMUSG00000021453	*Gadd45g*	2.32	2.89E-05
ENSMUSG00000052684	*Jun*	2.31	3.60E-08
ENSMUSG00000025804	*Ccr1*	2.31	7.47E-07
ENSMUSG00000076431	*Sox4*	2.30	3.35E-05
ENSMUSG00000028341	*Nr4a3*	2.29	3.75E-07
ENSMUSG00000031765	*Mt1*	2.29	8.38E-07
ENSMUSG00000028874	*Fgr*	2.29	1.64E-04
ENSMUSG00000064567	*SNORA38*	2.28	4.47E-03
ENSMUSG00000056708	*Ier5*	2.28	4.79E-07
ENSMUSG00000025225	*Nfkb2*	2.28	6.88E-07
ENSMUSG00000026068	*Il18rap*	2.28	2.53E-05
ENSMUSG00000074570	*Cass4*	2.28	9.48E-04
ENSMUSG00000035673	*Sbno2*	2.28	2.60E-06
ENSMUSG00000044562	*Rasip1*	2.26	5.20E-06
ENSMUSG00000042507	*C130039O16Rik*	2.25	1.47E-07
ENSMUSG00000089371	*Mir1938*	2.25	4.60E-03
ENSMUSG00000084497	*U6*	2.25	6.73E-03
ENSMUSG00000065592	*Mir145*	2.24	1.24E-04
ENSMUSG00000032000	*Birc3*	2.24	2.28E-07
ENSMUSG00000035373	*Ccl7*	2.24	1.25E-05
ENSMUSG00000022534	*Mefv*	2.23	4.98E-04

ENSMUSG00000002910	*Arrdc2*	2.23	6.85E-04
ENSMUSG00000022094	*Slc39a14*	2.22	5.61E-07
ENSMUSG00000015396	*Cd83*	2.22	2.87E-05
ENSMUSG00000020893	*Per1*	2.21	2.30E-06
ENSMUSG00000038059	*2010002N04Rik*	2.21	8.90E-05
ENSMUSG00000032661	*Oas3*	2.21	2.13E-03
ENSMUSG00000030852	*Tacc2*	2.20	4.73E-07
ENSMUSG00000022346	*Myc*	2.20	2.47E-05
ENSMUSG00000027894	*Slc6a17*	2.19	5.42E-03
ENSMUSG00000001379	*Apbb3*	2.18	1.80E-06
ENSMUSG00000043415	*Otud1*	2.18	5.03E-05
ENSMUSG00000025185	*Loxl4*	2.17	5.32E-05
ENSMUSG00000006642	*Tcf23*	2.16	7.59E-03
ENSMUSG00000055485	*9830001H06Rik*	2.14	2.35E-05
ENSMUSG00000026177	*Slc11a1*	2.14	4.47E-04
ENSMUSG00000034271	*Jdp2*	2.13	2.75E-06
ENSMUSG00000026358	*Rgs1*	2.13	2.75E-03
ENSMUSG00000035828	*Pim3*	2.13	2.56E-07
ENSMUSG00000001227	*Sema6b*	2.13	4.16E-04
ENSMUSG00000016024	*Lbp*	2.12	2.75E-06
ENSMUSG00000025650	*Col7a1*	2.11	1.90E-04
ENSMUSG00000078485	*Plekhn1*	2.11	8.90E-03
ENSMUSG00000018143	*Mafk*	2.11	3.04E-07
ENSMUSG00000030162	*Olr1*	2.11	3.13E-03
ENSMUSG00000011008	*Mcoln2*	2.11	4.29E-05
ENSMUSG00000049988	*Lrrc25*	2.10	3.63E-03
ENSMUSG00000020363	*Gfpt2*	2.10	4.26E-07
ENSMUSG00000019997	*Ctgf*	2.09	6.88E-07
ENSMUSG00000050721	*Plekho2*	2.08	3.09E-07
ENSMUSG00000029478	*Ncor2*	2.05	1.52E-06
ENSMUSG00000090582	*Gm17024*	2.05	2.71E-05
ENSMUSG00000003283	*Hck*	2.05	5.07E-04
ENSMUSG00000022893	*Adamts1*	2.05	1.19E-07
ENSMUSG00000000317	*Bcl6b*	2.04	1.09E-04
ENSMUSG00000078493	*A930039A15Rik*	2.04	2.21E-05
ENSMUSG00000032089	*Il10ra*	2.04	8.50E-06
ENSMUSG00000084894	*Gm13834*	2.04	4.64E-04
ENSMUSG00000021281	*Tnfaip2*	2.03	1.44E-06
ENSMUSG00000029228	*Lnx1*	2.03	3.28E-05
ENSMUSG00000023972	*Ptk7*	2.02	7.61E-03
ENSMUSG00000047180	*Neurl3*	2.02	3.35E-04
ENSMUSG00000020901	*Pik3r5*	2.01	3.74E-05
ENSMUSG00000049892	*Rasd1*	2.01	1.97E-03
ENSMUSG00000052911	*Lamb2*	2.00	1.47E-07

ENSMUSG00000073176	*Zfp449*	-2.06	1.26E-03
ENSMUSG00000046916	*Myct1*	-2.11	4.72E-03
ENSMUSG00000022491	*Glycam1*	-2.12	6.96E-04
ENSMUSG00000070168	*U3*	-2.12	1.41E-03
ENSMUSG00000065870	*Rnu3a*	-2.14	1.17E-05
ENSMUSG00000020335	*Zfp354b*	-2.17	7.31E-03
ENSMUSG00000065649	*Snora74a*	-2.21	5.48E-06
ENSMUSG00000065642	*Snora69*	-2.21	1.17E-04
ENSMUSG00000044609	*Gm9294*	-2.21	6.07E-03
ENSMUSG00000065359	*SNORD16*	-2.22	1.17E-05
ENSMUSG00000080622	*SNORA11*	-2.25	1.31E-04
ENSMUSG00000006586	*Runx1t1*	-2.26	2.29E-03
ENSMUSG00000070251	*U3*	-2.32	6.83E-04
ENSMUSG00000064837	*Snora75*	-2.34	1.27E-04
ENSMUSG00000084686	*SNORA19*	-2.38	1.21E-04
ENSMUSG00000065208	*SNORA4*	-2.43	6.13E-06
ENSMUSG00000064637	*Snora20*	-2.56	6.57E-05
ENSMUSG00000077611	*SNORA74*	-2.65	1.07E-06
ENSMUSG00000064620	*SNORA32*	-2.72	1.37E-04
ENSMUSG00000077756	*Snord90*	-2.76	3.04E-04
ENSMUSG00000045871	*Slitrk6*	-2.86	9.53E-03
ENSMUSG00000065041	*SNORA62*	-3.07	2.22E-07
ENSMUSG00000064451	*Snora23*	-3.09	1.35E-07
ENSMUSG00000088990	*SCARNA1*	-3.29	7.50E-04
ENSMUSG00000077167	*SNORA53*	-3.30	3.60E-08
ENSMUSG00000064917	*snoZ39*	-3.34	4.43E-07
ENSMUSG00000064792	*SNORA32*	-3.44	1.30E-03
ENSMUSG00000088895	*SNORA81*	-4.14	3.31E-03

[*] Genes that showed a $\log_2$ (fold change) > 2 and a FDR value of > 0.01 were defined as differentially expressed.
