# Supplementary figures and images for: Concurrent Host-Pathogen Transcriptional Responses in a Clostridium perfringens Murine Myonecrosis Infection
Source: mBio. 2018 Mar 27;9(2):e00473-18. doi: 10.1128/mBio.00473-18 (PMC5874911; doi:10.1128/mBio.00473-18)

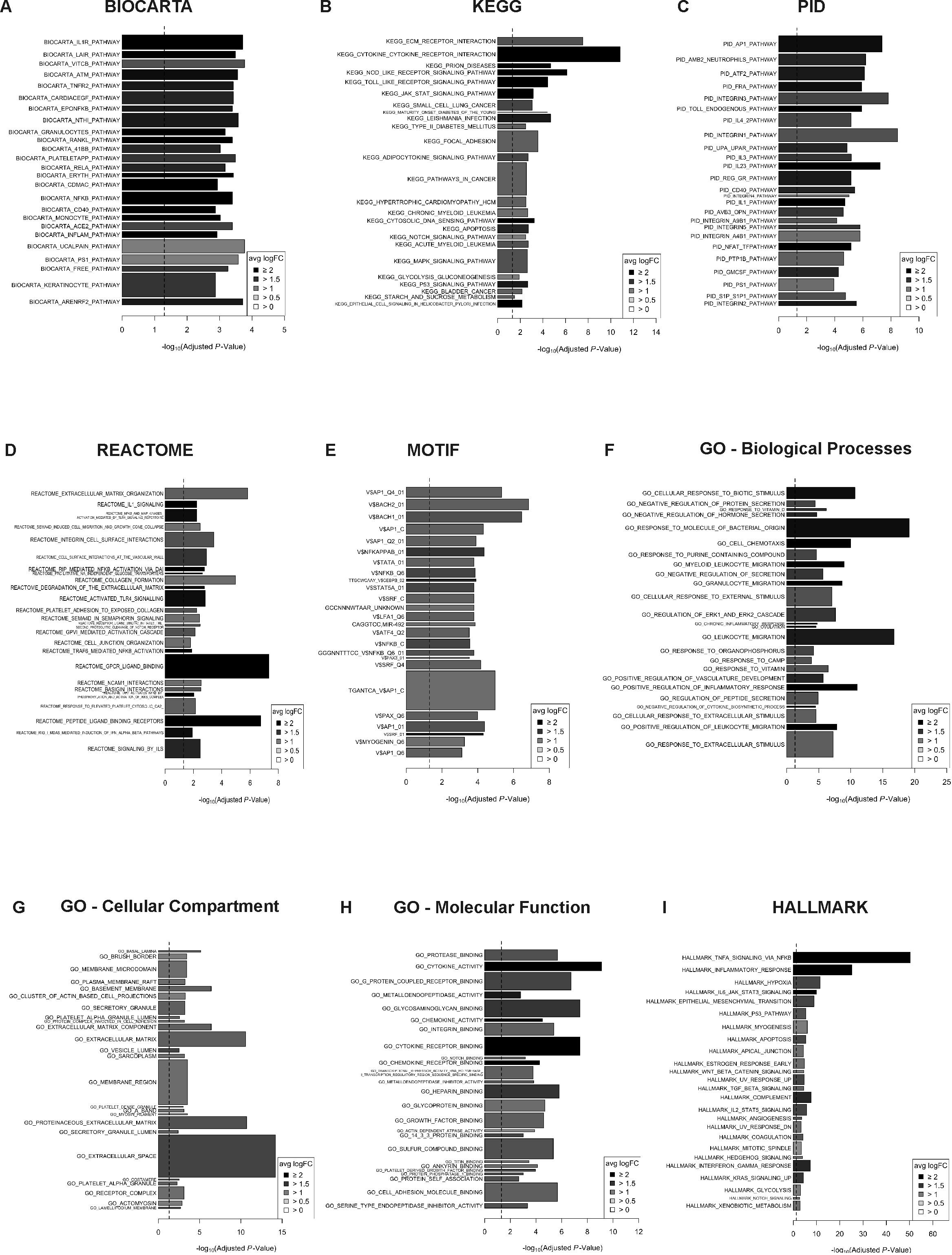

Supplement: FIG S1 [file mbo002183811sf1.tif]

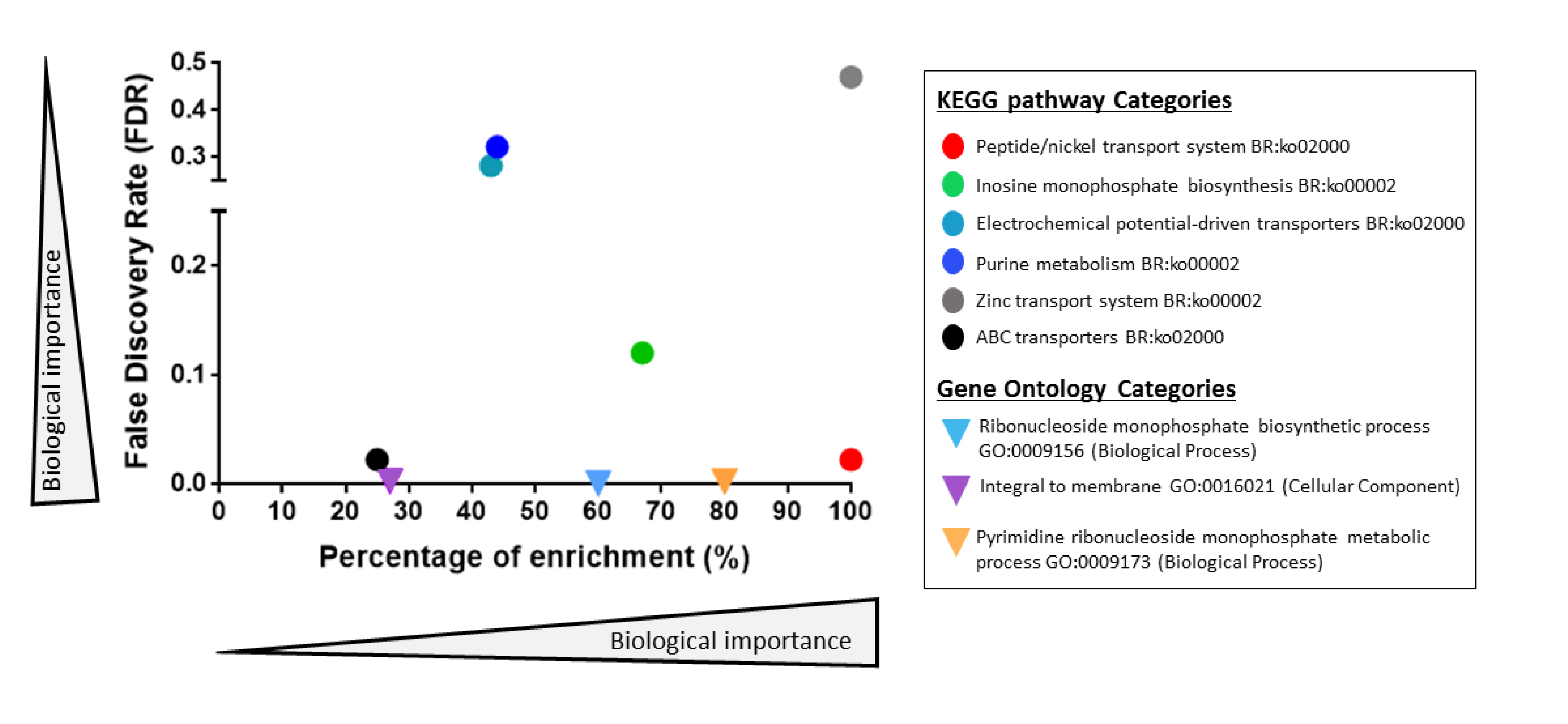

Supplement: FIG S2 [file mbo002183811sf2.tif]

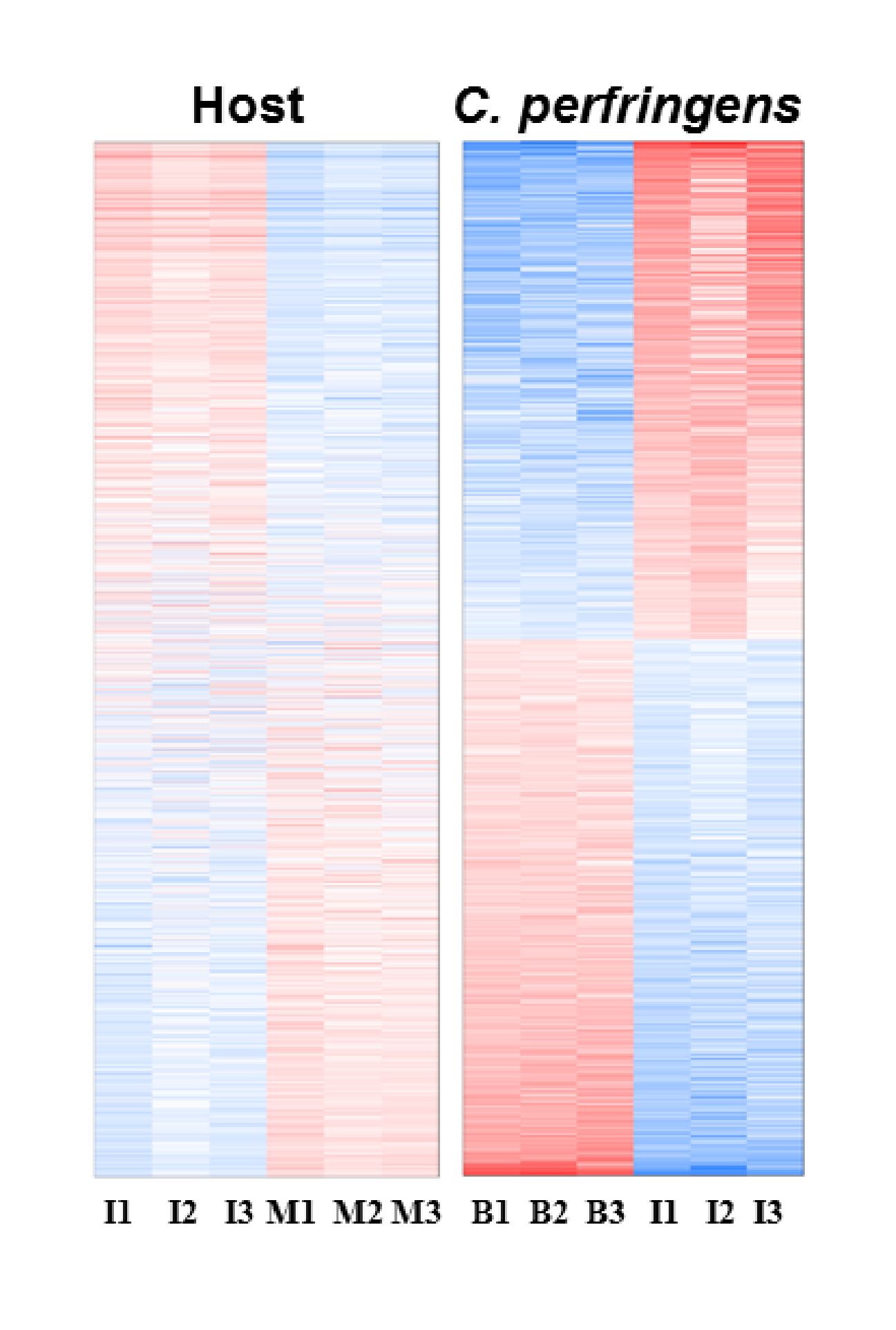

Supplement: FIG S3 [file mbo002183811sf3.tif]
