# Supplementary material for: Concurrent Host-Pathogen Transcriptional Responses in a Clostridium perfringens Murine Myonecrosis Infection
Source: mBio. 2018 Mar 27;9(2):e00473-18. doi: 10.1128/mBio.00473-18 (PMC5874911; doi:10.1128/mBio.00473-18)
Supplement: TABLE S2 [file mbo002183811st2.pdf]

TABLE S2 Gene set enrichment analyses of up-regulated murine genes in the infected mice compared to the mock-infected mice.

GO or KEGG ID	% of enrichment	*p* value
**Enriched GO Category**		
GO:0006954~inflammatory response	11.65	2.95E-15
GO:0006955~immune response	15.66	5.35E-15
GO:0009611~response to wounding	13.25	6.98E-14
GO:0006952~defense response	14.06	2.78E-12
GO:0042330~taxis	7.23	5.08E-10
GO:0006935~chemotaxis	7.23	5.08E-10
GO:0001817~regulation of cytokine production	6.83	2.95E-07
GO:0007610~behavior	10.44	1.75E-06
GO:0001819~positive regulation of cytokine production	4.82	2.11E-06
GO:0050900~leukocyte migration	4.02	1.51E-05
GO:0009891~positive regulation of biosynthetic process	11.65	1.60E-05
GO:0007626~locomotory behavior	7.63	2.12E-05
GO:0030595~leukocyte chemotaxis	3.21	1.45E-04
GO:0060326~cell chemotaxis	3.21	1.45E-04
GO:0031328~positive regulation of cellular biosynthetic process	10.84	2.08E-04
GO:0045944~positive regulation of transcription from RNA polymerase II promoter	8.43	4.64E-04
GO:0002237~response to molecule of bacterial origin	3.61	7.95E-04
GO:0009617~response to bacterium	5.62	8.13E-04
GO:0045893~positive regulation of transcription, DNA-dependent	8.84	1.22E-03
GO:0010557~positive regulation of macromolecule biosynthetic process	10.04	1.35E-03
GO:0051254~positive regulation of RNA metabolic process	8.84	1.38E-03
GO:0051173~positive regulation of nitrogen compound metabolic process	9.64	4.20E-03
GO:0002685~regulation of leukocyte migration	2.01	4.88E-03
GO:0006357~regulation of transcription from RNA polymerase II promoter	10.44	5.67E-03
GO:0044092~negative regulation of molecular function	4.82	5.92E-03
GO:0051240~positive regulation of multicellular organismal process	5.22	7.78E-03
GO:0043410~positive regulation of MAPKKK cascade	3.21	8.03E-03
GO:0010604~positive regulation of macromolecule metabolic process	10.44	9.15E-03
GO:0045941~positive regulation of transcription	8.84	9.99E-03
GO:0007155~cell adhesion	9.64	1.22E-02
GO:0022610~biological adhesion	9.64	1.26E-02
GO:0010628~positive regulation of gene expression	8.84	1.51E-02
GO:0032496~response to lipopolysaccharide	2.81	2.77E-02
GO:0050830~defense response to Gram-positive bacterium	2.41	2.83E-02

GO:0045935~positive regulation of nucleobase, nucleoside, nucleotide and nucleic acid metabolic process	8.84	2.94E-02
GO:0042742~defense response to bacterium	4.02	4.64E-02
GO:0002687~positive regulation of leukocyte migration	1.61	4.97E-02
GO:0032680~regulation of tumor necrosis factor production	2.41	6.48E-02
GO:0002684~positive regulation of immune system process	5.22	8.17E-02
GO:0032760~positive regulation of tumor necrosis factor production	2.01	8.78E-02
GO:0051051~negative regulation of transport	3.21	1.38E-01
GO:0030593~neutrophil chemotaxis	2.01	1.50E-01
GO:0046888~negative regulation of hormone secretion	2.01	1.50E-01
GO:0042325~regulation of phosphorylation	6.02	1.50E-01
GO:0042127~regulation of cell proliferation	8.43	1.94E-01
GO:0019220~regulation of phosphate metabolic process	6.02	2.23E-01
GO:0051174~regulation of phosphorus metabolic process	6.02	2.23E-01
GO:0046883~regulation of hormone secretion	2.41	2.72E-01
GO:0042981~regulation of apoptosis	8.43	2.80E-01
GO:0030335~positive regulation of cell migration	2.41	3.10E-01
GO:0043067~regulation of programmed cell death	8.43	3.31E-01
GO:0010941~regulation of cell death	8.43	3.55E-01
GO:0008284~positive regulation of cell proliferation	5.62	4.50E-01
GO:0051674~localization of cell	5.62	4.50E-01
GO:0048870~cell motility	5.62	4.50E-01
GO:0010740~positive regulation of protein kinase cascade	3.21	4.89E-01
GO:0051272~positive regulation of cell motion	2.41	5.08E-01
GO:0002221~pattern recognition receptor signaling pathway	1.61	5.64E-01
GO:0032088~negative regulation of NF-kappaB transcription factor activity	1.61	5.64E-01
GO:0043408~regulation of MAPKKK cascade	3.21	6.86E-01
GO:0010033~response to organic substance	7.63	7.10E-01
GO:0040017~positive regulation of locomotion	2.41	7.11E-01
GO:0010627~regulation of protein kinase cascade	4.02	7.39E-01
GO:0032494~response to peptidoglycan	1.61	7.66E-01
GO:0006915~apoptosis	7.23	7.71E-01
GO:0045765~regulation of angiogenesis	2.41	7.90E-01
GO:0051046~regulation of secretion	3.61	8.05E-01
GO:0012501~programmed cell death	7.23	9.35E-01
GO:0051048~negative regulation of secretion	2.01	9.80E-01
GO:0002758~innate immune response-activating signal transduction	1.61	1.01E+00
GO:0016477~cell migration	4.82	1.32E+00
GO:0006953~acute-phase response	2.01	1.48E+00
GO:0051100~negative regulation of binding	2.01	1.48E+00
GO:0001568~blood vessel development	4.82	1.50E+00
GO:0009967~positive regulation of signal transduction	4.02	1.56E+00
GO:0001775~cell activation	4.82	1.61E+00
GO:0006928~cell motion	6.02	1.63E+00

GO:0002218~activation of innate immune response	1.61	1.63E+00
GO:0002263~cell activation during immune response	2.01	1.67E+00
GO:0002366~leukocyte activation during immune response	2.01	1.67E+00
GO:0001944~vasculature development	4.82	1.83E+00
GO:0032675~regulation of interleukin-6 production	2.01	1.89E+00
GO:0051726~regulation of cell cycle	4.42	1.94E+00
GO:0008219~cell death	7.23	2.00E+00
GO:0042832~defense response to protozoan	1.20	2.05E+00
GO:0016265~death	7.23	2.57E+00
GO:0051090~regulation of transcription factor activity	2.41	2.72E+00
GO:0007242~intracellular signaling cascade	10.44	2.89E+00
GO:0045089~positive regulation of innate immune response	2.01	2.94E+00
GO:0010647~positive regulation of cell communication	4.02	2.98E+00
GO:0001892~embryonic placenta development	2.41	3.15E+00
GO:0051098~regulation of binding	2.81	3.29E+00
GO:0070391~response to lipoteichoic acid	1.20	3.37E+00
GO:0032755~positive regulation of interleukin-6 production	1.61	3.47E+00
GO:0030334~regulation of cell migration	2.81	3.67E+00
GO:0060341~regulation of cellular localization	3.61	4.06E+00
GO:0043122~regulation of I-kappaB kinase/NF-kappaB cascade	2.01	4.33E+00
GO:0051252~regulation of RNA metabolic process	14.46	4.66E+00
GO:0033138~positive regulation of peptidyl-serine phosphorylation	1.20	4.97E+00
GO:0008283~cell proliferation	4.42	5.48E+00
GO:0001816~cytokine production	2.01	5.62E+00
GO:0043086~negative regulation of catalytic activity	2.81	5.80E+00
GO:0043433~negative regulation of transcription factor activity	1.61	6.19E+00
GO:0032101~regulation of response to external stimulus	2.81	6.37E+00
GO:0006355~regulation of transcription, DNA-dependent	14.06	6.60E+00
GO:0051094~positive regulation of developmental process	4.02	6.69E+00
GO:0001562~response to protozoan	1.20	6.82E+00
GO:0070230~positive regulation of lymphocyte apoptosis	1.20	6.82E+00
GO:0050766~positive regulation of phagocytosis	1.61	7.02E+00
GO:0045088~regulation of innate immune response	2.01	7.13E+00
GO:0051101~regulation of DNA binding	2.41	7.19E+00
GO:0051270~regulation of cell motion	2.81	7.63E+00
GO:0002521~leukocyte differentiation	3.21	7.67E+00
GO:0001818~negative regulation of cytokine production	1.61	7.90E+00
GO:0040012~regulation of locomotion	2.81	8.68E+00
GO:0050764~regulation of phagocytosis	1.61	8.84E+00
GO:0006909~phagocytosis	2.01	8.87E+00
GO:0048584~positive regulation of response to stimulus	3.61	9.45E+00
GO:0043392~negative regulation of DNA binding	1.61	9.84E+00
GO:0033673~negative regulation of kinase activity	2.01	1.02E+01
GO:0006469~negative regulation of protein kinase activity	2.01	1.02E+01

GO:0051348~negative regulation of transferase activity	2.01	1.16E+01
GO:0002460~adaptive immune response based on somatic recombination of immune receptors built from immunoglobulin superfamily domains	2.41	1.20E+01
GO:0002250~adaptive immune response	2.41	1.20E+01
GO:0050865~regulation of cell activation	3.21	1.24E+01
GO:0043066~negative regulation of apoptosis	4.02	1.32E+01
GO:0048514~blood vessel morphogenesis	3.61	1.33E+01
GO:0001932~regulation of protein amino acid phosphorylation	2.81	1.33E+01
GO:0001890~placenta development	2.41	1.37E+01
GO:0042116~macrophage activation	1.20	1.37E+01
GO:0031349~positive regulation of defense response	2.01	1.47E+01
GO:0043069~negative regulation of programmed cell death	4.02	1.48E+01
GO:0060548~negative regulation of cell death	4.02	1.50E+01
GO:0002252~immune effector process	2.81	1.59E+01
GO:0033135~regulation of peptidyl-serine phosphorylation	1.20	1.64E+01
GO:0032642~regulation of chemokine production	1.20	1.64E+01
GO:0043068~positive regulation of programmed cell death	4.02	1.70E+01
GO:0010942~positive regulation of cell death	4.02	1.78E+01
GO:0009968~negative regulation of signal transduction	3.21	1.91E+01
GO:0051050~positive regulation of transport	2.81	1.93E+01
GO:0045807~positive regulation of endocytosis	1.61	1.97E+01
GO:0001525~angiogenesis	2.81	1.99E+01
GO:0042035~regulation of cytokine biosynthetic process	2.01	2.11E+01
GO:0060711~labyrinthine layer development	1.61	2.12E+01
GO:0050731~positive regulation of peptidyl-tyrosine phosphorylation	1.61	2.12E+01
GO:0006873~cellular ion homeostasis	4.02	2.15E+01
GO:0032651~regulation of interleukin-1 beta production	1.20	2.21E+01
GO:0045892~negative regulation of transcription, DNA-dependent	4.42	2.22E+01
GO:0051253~negative regulation of RNA metabolic process	4.42	2.31E+01
GO:0050867~positive regulation of cell activation	2.41	2.36E+01
GO:0030198~extracellular matrix organization	2.41	2.36E+01
GO:0001701~in utero embryonic development	4.02	2.42E+01
GO:0055082~cellular chemical homeostasis	4.02	2.46E+01
GO:0032652~regulation of interleukin-1 production	1.20	2.50E+01
GO:0070228~regulation of lymphocyte apoptosis	1.20	2.50E+01
GO:0002286~T cell activation during immune response	1.20	2.50E+01
GO:0007159~leukocyte adhesion	1.20	2.50E+01
GO:0045449~regulation of transcription	18.07	2.75E+01
GO:0045859~regulation of protein kinase activity	3.21	2.77E+01
GO:0010648~negative regulation of cell communication	3.21	2.77E+01
GO:0032655~regulation of interleukin-12 production	1.20	2.81E+01
GO:0010558~negative regulation of macromolecule biosynthetic process	5.22	2.81E+01

GO:0000122~negative regulation of transcription from RNA polymerase II promoter	3.61	2.86E+01
GO:0045087~innate immune response	2.41	2.87E+01
GO:0048534~hemopoietic or lymphoid organ development	4.02	3.11E+01
GO:0046330~positive regulation of JNK cascade	1.20	3.12E+01
GO:0070304~positive regulation of stress-activated protein kinase signaling pathway	1.20	3.12E+01
GO:0007169~transmembrane receptor protein tyrosine kinase signaling pathway	3.21	3.13E+01
GO:0043549~regulation of kinase activity	3.21	3.13E+01
GO:0007243~protein kinase cascade	3.61	3.14E+01
GO:0031327~negative regulation of cellular biosynthetic process	5.22	3.31E+01
GO:0030217~T cell differentiation	2.01	3.43E+01
GO:0045766~positive regulation of angiogenesis	1.20	3.43E+01
GO:0060713~labyrinthine layer morphogenesis	1.20	3.43E+01
GO:0009620~response to fungus	1.20	3.43E+01
GO:0006468~protein amino acid phosphorylation	6.83	3.44E+01
GO:0048568~embryonic organ development	3.61	3.46E+01
GO:0009890~negative regulation of biosynthetic process	5.22	3.48E+01
GO:0051338~regulation of transferase activity	3.21	3.65E+01
GO:0042110~T cell activation	2.41	3.70E+01
GO:0060674~placenta blood vessel development	1.20	3.74E+01
GO:0050801~ion homeostasis	4.02	3.76E+01
GO:0019725~cellular homeostasis	4.42	3.85E+01
GO:0002268~follicular dendritic cell differentiation	0.80	3.87E+01
GO:0007263~nitric oxide mediated signal transduction	0.80	3.87E+01
GO:0002266~follicular dendritic cell activation	0.80	3.87E+01
GO:0070163~regulation of adiponectin secretion	0.80	3.87E+01
GO:0002688~regulation of leukocyte chemotaxis	0.80	3.87E+01
GO:0002752~cell surface pattern recognition receptor signaling pathway	0.80	3.87E+01
GO:0045835~negative regulation of meiosis	0.80	3.87E+01
GO:0042503~tyrosine phosphorylation of Stat3 protein	0.80	3.87E+01
GO:0002520~immune system development	4.02	3.88E+01
GO:0043065~positive regulation of apoptosis	3.61	3.88E+01
GO:0002526~acute inflammatory response	2.01	4.04E+01
GO:0045428~regulation of nitric oxide biosynthetic process	1.20	4.05E+01
GO:0002285~lymphocyte activation during immune response	1.20	4.05E+01
GO:0046632~alpha-beta T cell differentiation	1.20	4.05E+01
GO:0030097~hemopoiesis	3.61	4.08E+01
GO:0002757~immune response-activating signal transduction	1.61	4.25E+01
GO:0051130~positive regulation of cellular component organization	2.41	4.28E+01
GO:0031399~regulation of protein modification process	2.81	4.37E+01
GO:0010605~negative regulation of macromolecule metabolic process	5.62	4.40E+01
GO:0043281~regulation of caspase activity	1.61	4.59E+01

GO:0052548~regulation of endopeptidase activity	1.61	4.59E+01
GO:0010553~negative regulation of specific transcription from RNA polymerase II promoter	1.20	4.67E+01
GO:0052547~regulation of peptidase activity	1.61	4.77E+01
GO:0032147~activation of protein kinase activity	1.61	4.77E+01
GO:0045637~regulation of myeloid cell differentiation	1.61	4.77E+01
GO:0030100~regulation of endocytosis	1.61	4.94E+01
GO:0002764~immune response-regulating signal transduction	1.61	4.94E+01
GO:0007611~learning or memory	2.01	5.02E+01
GO:0045321~leukocyte activation	3.21	5.05E+01
GO:0048878~chemical homeostasis	4.42	5.06E+01
GO:0050730~regulation of peptidyl-tyrosine phosphorylation	1.61	5.11E+01
GO:0008228~opsonization	0.80	5.20E+01
GO:0007260~tyrosine phosphorylation of STAT protein	0.80	5.20E+01
GO:0002238~response to molecule of fungal origin	0.80	5.20E+01
GO:0060708~spongiotrophoblast differentiation	0.80	5.20E+01
GO:0010572~positive regulation of platelet activation	0.80	5.20E+01
GO:0010543~regulation of platelet activation	0.80	5.20E+01
GO:0010273~detoxification of copper ion	0.80	5.20E+01
GO:0002367~cytokine production during immune response	0.80	5.20E+01
GO:0046631~alpha-beta T cell activation	1.20	5.26E+01
GO:0032582~negative regulation of gene-specific transcription	1.20	5.26E+01
GO:0031099~regeneration	1.20	5.26E+01
GO:0043009~chordate embryonic development	4.82	5.32E+01
GO:0016481~negative regulation of transcription	4.42	5.49E+01
GO:0009792~embryonic development ending in birth or egg hatching	4.82	5.51E+01
GO:0007167~enzyme linked receptor protein signaling pathway	3.61	5.55E+01
GO:0019221~cytokine-mediated signaling pathway	1.61	5.61E+01
GO:0001934~positive regulation of protein amino acid phosphorylation	1.61	5.61E+01
GO:0050778~positive regulation of immune response	2.41	5.66E+01
GO:0051092~positive regulation of NF-kappaB transcription factor activity	1.20	5.82E+01
GO:0050673~epithelial cell proliferation	1.20	6.08E+01
GO:0046635~positive regulation of alpha-beta T cell activation	1.20	6.08E+01
GO:0042327~positive regulation of phosphorylation	1.61	6.24E+01
GO:0002281~macrophage activation during immune response	0.80	6.24E+01
GO:0010574~regulation of vascular endothelial growth factor production	0.80	6.24E+01
GO:0070423~nucleotide-binding oligomerization domain containing signaling pathway	0.80	6.24E+01
GO:0002220~innate immune response activating cell surface receptor signaling pathway	0.80	6.24E+01
GO:0070431~nucleotide-binding oligomerization domain containing 2 signaling pathway	0.80	6.24E+01
GO:0032695~negative regulation of interleukin-12 production	0.80	6.24E+01
GO:0001660~fever	0.80	6.24E+01

GO:0033627~cell adhesion mediated by integrin	0.80	6.24E+01
GO:0006927~transformed cell apoptosis	0.80	6.24E+01
GO:0010575~positive regulation vascular endothelial growth factor production	0.80	6.24E+01
GO:0016337~cell-cell adhesion	3.21	6.27E+01
GO:0032270~positive regulation of cellular protein metabolic process	2.01	6.33E+01
GO:0051249~regulation of lymphocyte activation	2.41	6.41E+01
GO:0016310~phosphorylation	6.83	6.44E+01
GO:0045937~positive regulation of phosphate metabolic process	1.61	6.54E+01
GO:0010562~positive regulation of phosphorus metabolic process	1.61	6.54E+01
GO:0045934~negative regulation of nucleobase, nucleoside, nucleotide and nucleic acid metabolic process	4.42	6.73E+01
GO:0043062~extracellular structure organization	2.41	6.85E+01
GO:0051172~negative regulation of nitrogen compound metabolic process	4.42	6.97E+01
GO:0050708~regulation of protein secretion	1.20	7.04E+01
GO:0007422~peripheral nervous system development	1.20	7.04E+01
GO:0032495~response to muramyl dipeptide	0.80	7.06E+01
GO:0042093~T-helper cell differentiation	0.80	7.06E+01
GO:0002294~CD4-positive, alpha-beta T cell differentiation during immune response	0.80	7.06E+01
GO:0006828~manganese ion transport	0.80	7.06E+01
GO:0002904~positive regulation of B cell apoptosis	0.80	7.06E+01
GO:0030003~cellular cation homeostasis	2.41	7.10E+01
GO:0032583~regulation of gene-specific transcription	1.61	7.23E+01
GO:0002694~regulation of leukocyte activation	2.41	7.26E+01
GO:0051247~positive regulation of protein metabolic process	2.01	7.28E+01
GO:0010629~negative regulation of gene expression	4.42	7.43E+01
GO:0044093~positive regulation of molecular function	3.61	7.43E+01
GO:0032649~regulation of interferon-gamma production	1.20	7.46E+01
GO:0006793~phosphorus metabolic process	7.63	7.57E+01
GO:0006796~phosphate metabolic process	7.63	7.57E+01
GO:0043123~positive regulation of I-kappaB kinase/NF-kappaB cascade	1.20	7.65E+01
GO:0002467~germinal center formation	0.80	7.70E+01
GO:0001878~response to yeast	0.80	7.70E+01
GO:0045123~cellular extravasation	0.80	7.70E+01
GO:0021783~preganglionic parasympathetic nervous system development	0.80	7.70E+01
GO:0002292~T cell differentiation during immune response	0.80	7.70E+01
GO:0002293~alpha-beta T cell differentiation during immune response	0.80	7.70E+01
GO:0060707~trophoblast giant cell differentiation	0.80	7.70E+01
GO:0030098~lymphocyte differentiation	2.01	7.74E+01
GO:0046634~regulation of alpha-beta T cell activation	1.20	7.83E+01
GO:0002573~myeloid leukocyte differentiation	1.20	7.83E+01
GO:0051091~positive regulation of transcription factor activity	1.20	7.83E+01
GO:0042692~muscle cell differentiation	2.01	7.99E+01

GO:0002274~myeloid leukocyte activation	1.20	8.00E+01
GO:0051222~positive regulation of protein transport	1.20	8.16E+01
GO:0050716~positive regulation of interleukin-1 secretion	0.80	8.20E+01
GO:0050706~regulation of interleukin-1 beta secretion	0.80	8.20E+01
GO:0002456~T cell mediated immunity	0.80	8.20E+01
GO:0032731~positive regulation of interleukin-1 beta production	0.80	8.20E+01
GO:0031649~heat generation	0.80	8.20E+01
GO:0048486~parasympathetic nervous system development	0.80	8.20E+01
GO:0060664~epithelial cell proliferation involved in salivary gland morphogenesis	0.80	8.20E+01
GO:0045073~regulation of chemokine biosynthetic process	0.80	8.20E+01
GO:0050994~regulation of lipid catabolic process	0.80	8.20E+01
GO:0002287~alpha-beta T cell activation during immune response	0.80	8.20E+01
GO:0046688~response to copper ion	0.80	8.20E+01
GO:0046627~negative regulation of insulin receptor signaling pathway	0.80	8.20E+01
GO:0043901~negative regulation of multi-organism process	0.80	8.20E+01
GO:0002224~toll-like receptor signaling pathway	0.80	8.20E+01
GO:0050704~regulation of interleukin-1 secretion	0.80	8.20E+01
GO:0006882~cellular zinc ion homeostasis	0.80	8.20E+01
GO:0050718~positive regulation of interleukin-1 beta secretion	0.80	8.20E+01
GO:0043903~regulation of symbiosis, encompassing mutualism through parasitism	0.80	8.20E+01
GO:0046697~decidualization	0.80	8.20E+01
GO:0032732~positive regulation of interleukin-1 production	0.80	8.20E+01
GO:0002449~lymphocyte mediated immunity	1.61	8.25E+01
GO:0044421~extracellular region part	14.46	6.14E-12
GO:0005615~extracellular space	11.24	7.60E-11
GO:0005576~extracellular region	18.47	1.06E-07
GO:0009986~cell surface	5.22	3.25E-04
GO:0005578~proteinaceous extracellular matrix	4.42	3.24E-03
GO:0009897~external side of plasma membrane	3.61	3.53E-03
GO:0031012~extracellular matrix	4.42	4.28E-03
GO:0005886~plasma membrane	20.08	5.89E-03
GO:0030659~cytoplasmic vesicle membrane	2.01	2.10E-02
GO:0031982~vesicle	5.22	2.33E-02
GO:0044433~cytoplasmic vesicle part	2.01	2.79E-02
GO:0012506~vesicle membrane	2.01	2.98E-02
GO:0030141~secretory granule	2.01	5.32E-02
GO:0005604~basement membrane	1.61	5.82E-02
GO:0030670~phagocytic vesicle membrane	0.80	7.03E-02
GO:0045121~membrane raft	1.61	8.35E-02
GO:0031410~cytoplasmic vesicle	4.42	8.79E-02
GO:0005125~cytokine activity	6.83	3.02E-09
GO:0030246~carbohydrate binding	7.63	3.26E-07
GO:0008009~chemokine activity	3.21	6.84E-07

GO:0042379~chemokine receptor binding	3.21	8.25E-07
GO:0030247~polysaccharide binding	4.02	6.28E-05
GO:0001871~pattern binding	4.02	6.28E-05
GO:0005149~interleukin-1 receptor binding	1.61	6.43E-04
GO:0005529~sugar binding	4.02	8.36E-04
GO:0004222~metalloendopeptidase activity	3.21	9.37E-04
GO:0005539~glycosaminoglycan binding	3.21	9.37E-04
GO:0019955~cytokine binding	2.81	1.26E-03
GO:0043565~sequence-specific DNA binding	7.23	1.55E-03
GO:0008083~growth factor activity	3.21	3.17E-03
GO:0030528~transcription regulator activity	11.65	3.58E-03
GO:0003700~transcription factor activity	8.43	4.51E-03
GO:0008201~heparin binding	2.41	5.47E-03
GO:0004896~cytokine receptor activity	2.01	6.61E-03
GO:0008237~metallopeptidase activity	3.21	1.42E-02
GO:0008528~peptide receptor activity, G-protein coupled	2.41	1.72E-02
GO:0001653~peptide receptor activity	2.41	1.72E-02
GO:0015294~solute:cation symporter activity	2.01	1.75E-02
GO:0046983~protein dimerization activity	4.42	1.81E-02
GO:0015293~symporter activity	2.41	3.44E-02
GO:0004950~chemokine receptor activity	1.20	3.57E-02
GO:0019956~chemokine binding	1.20	3.87E-02
GO:0001872~zymosan binding	0.80	4.03E-02
GO:0046915~transition metal ion transmembrane transporter activity	1.20	4.19E-02
GO:0004857~enzyme inhibitor activity	3.21	4.86E-02
GO:0005384~manganese ion transmembrane transporter activity	0.80	5.34E-02
GO:0003677~DNA binding	13.25	5.98E-02
GO:0042277~peptide binding	2.41	6.78E-02
GO:0004866~endopeptidase inhibitor activity	2.41	6.92E-02
GO:0015082~di-, tri-valent inorganic cation transmembrane transporter activity	1.20	7.01E-02
GO:0019838~growth factor binding	1.61	7.50E-02
GO:0046982~protein heterodimerization activity	2.01	8.34E-02
GO:0004908~interleukin-1 receptor activity	0.80	9.16E-02
GO:0019966~interleukin-1 binding	0.80	9.16E-02
GO:0030414~peptidase inhibitor activity	2.41	9.29E-02
**Enriched KEGG Categories**		
mmu04621:NOD-like receptor signaling pathway	5.22	1.77E-10
mmu04060:Cytokine-cytokine receptor interaction	8.43	1.14E-09
mmu04620:Toll-like receptor signaling pathway	5.22	4.65E-08
mmu04010:MAPK signaling pathway	7.23	8.61E-07
mmu04640:Hematopoietic cell lineage	4.02	7.08E-06
mmu04630:Jak-STAT signaling pathway	4.82	2.95E-05
mmu04062:Chemokine signaling pathway	5.22	3.18E-05
mmu05200:Pathways in cancer	6.43	1.84E-04

mmu05222:Small cell lung cancer	2.81	2.41E-03
mmu04012:ErbB signaling pathway	2.41	1.31E-02
mmu04210:Apoptosis	2.41	1.31E-02
mmu05332:Graft-versus-host disease	2.01	1.41E-02
mmu04510:Focal adhesion	3.61	1.45E-02
mmu05020:Prion diseases	1.61	1.86E-02
mmu04115:p53 signaling pathway	2.01	2.51E-02
mmu05219:Bladder cancer	1.61	3.00E-02
mmu04960:Aldosterone-regulated sodium reabsorption	1.61	3.00E-02
mmu04660:T cell receptor signaling pathway	2.41	4.19E-02
mmu04930:Type II diabetes mellitus	1.61	4.44E-02
mmu04512:ECM-receptor interaction	2.01	4.51E-02
mmu04623:Cytosolic DNA-sensing pathway	1.61	5.91E-02
mmu04666:Fc gamma R-mediated phagocytosis	2.01	7.40E-02
mmu04940:Type I diabetes mellitus	1.61	8.17E-02
mmu04920:Adipocytokine signaling pathway	1.61	9.42E-02
