# Supplementary material for: Concurrent Host-Pathogen Transcriptional Responses in a Clostridium perfringens Murine Myonecrosis Infection
Source: mBio. 2018 Mar 27;9(2):e00473-18. doi: 10.1128/mBio.00473-18 (PMC5874911; doi:10.1128/mBio.00473-18)
Supplement: TABLE S3 [file mbo002183811st3.pdf]

TABLE S3 Differentially expressed genes in *C. perfringens* cells grown *in vivo* compared to *in vitro*.

Locus Tag[a]	Log_2 (fold Change)	FDR	Gene[b]	Putative Product
CPE0489	7.70	8.32E-07	*pscX*	flippase
CPE1182	6.82	4.37E-05		aspartate carbamoyltransferase regulatory subunit
CPE2250	6.25	7.28E-05		hypothetical protein
CPE0690	6.15	4.66E-06	*argH*	argininosuccinate lyase
CPE1183	5.85	3.79E-06	*pyrB*	aspartate carbamoyltransferase
CPE1616	5.37	1.27E-04		hypothetical protein
CPE2274	5.31	3.22E-05	*narK*	nitrate extrusion protein
CPE2563	5.30	1.01E-03		Ser-type protease
CPE1858	5.26	1.31E-06		cell division protein FtsQ
PCP12	5.21	3.22E-05		hypothetical protein
CPE2573	5.17	1.49E-04	*carA*	carbamoyl phosphate synthase small subunit
CPE1177	5.09	4.92E-06	*pyrE*	orotate phosphoribosyltransferase
CPE2135	5.04	2.51E-05		hypothetical protein
CPE0747	5.03	4.66E-05		hypothetical protein
CPE0155	5.02	2.57E-04		hypothetical protein
CPE1181	4.93	1.39E-05	*pyrC*	dihydroorotase, multifunctional complex type
CPE1410	4.92	7.22E-05		Na-translocating NADH-quinone reductase Nqr2 subunit
CPE1480	4.71	1.02E-04		sulfate permease, SulP family
PCP10	4.64	2.47E-04		transposase
CPE0465	4.53	2.46E-04		hypothetical protein
CPE0369	4.52	9.61E-05		hypothetical protein
CPE0276	4.44	2.59E-03		hydrogenase
CPE2572	4.42	7.08E-06	*carB*	carbamoyl phosphate synthase large subunit
CPE0713	4.40	1.28E-04		hypothetical protein
CPE2223	4.39	2.32E-04		mannosyltransferase
CPE1388	4.36	1.64E-03		hypothetical protein
CPE0809	4.36	4.12E-07		vancomycin high temperature exclusion protein
CPE0156	4.30	1.24E-03		surface protein
CPE2359	4.30	8.77E-07	*rpsD*	30S ribosomal protein S4
CPE0382	4.28	9.08E-04		autolytic lysozyme
CPE1609	4.28	4.40E-04		hypothetical protein
CPE1799	4.27	2.45E-03		FAD dependent oxidoreductase
CPE2179	4.27	1.98E-04		sporulation protein YyaC
CPE0066	4.25	4.14E-03		amylopullulanase
CPE2157	4.19	9.93E-03		PTS system, glucose-specific IIBC component
CPE1903	4.17	1.94E-04		small acid-soluble spore protein beta
PCP20	4.16	3.80E-04		hypothetical protein
CPE0227	4.15	1.93E-03		ABC transporter

CPE0965	4.13	3.10E-04		GGDEF domain protein
CPE1359	4.09	3.10E-04		hypothetical protein
CPE1133	4.08	5.36E-03		choline binding protein
CPE1809	4.08	3.05E-04	*spoIIM*	stage II sporulation protein M
CPE2046	4.08	5.97E-03	*spoVAD*	stage V sporulation protein AD
CPE0648	4.08	3.56E-03	*gerKA*	spore germination protein KA
CPE1178	4.07	8.34E-05	*pyrD*	dihydroorotate dehydrogenase 1B
CPE1138	4.07	6.39E-04		hypothetical protein
CPE1559	4.05	2.34E-04		diapophytoene dehydrogenase
CPE1586	4.05	2.69E-04		hypothetical protein
SR93	4.02	5.02E-04		Region between CPE2632 and CPE2633
CPE1769	4.00	1.71E-03	*spoVD*	stage V sporulation protein D
CPE0192	4.00	6.91E-04	*cbiM*	cobalt transport protein CbiM
CPE0241	3.99	1.13E-03		flagellar motor protein MotB
CPE1125	3.98	7.76E-03		hypothetical protein
CPE1377	3.97	1.09E-06		hypothetical protein
CPE2561	3.95	9.11E-03		Ser-type protease
CPE1095	3.94	1.15E-03		integrase/recombinase
CPE2213	3.90	4.68E-04	*cphA*	cyanophycin synthetase
PCP05	3.89	2.75E-03		transposase
CPE0797	3.88	1.53E-04		PAP2 family protein
CPE0223	3.88	3.31E-03		ferrichrome ABC transporter
CPE0938	3.88	1.08E-03		putative sulfite oxidase subunit YedZ
CPE0117	3.87	4.40E-06		hypothetical protein
CPE1648	3.87	5.17E-04	*spoVB*	stage V sporulation protein B
CPE0643	3.83	1.92E-06		hypothetical protein
CPE0397	3.80	8.45E-06	*uapC*	transporter
CPE2558	3.79	2.76E-03		hypothetical protein
CPE1829	3.78	5.65E-03	*spoIIIAE*	stage III sporulation protein AE
CPE0517	3.77	1.36E-03		von Willebrand factor A
CPE1620	3.77	7.92E-04		hypothetical protein
CPE2611	3.76	6.30E-03		peptidase, M23/M37 family
CPE0601	3.75	1.33E-03		amino acid ABC transporter
CPE0728	3.75	1.68E-03		short chain dehydrogenase
CPE2041	3.74	2.48E-03	*gpr*	germination protease
CPE1578	3.74	4.74E-03		ABC transporter
CPE1843	3.74	6.30E-04	*pilC*	fimbrial assembly protein
CPE2458	3.73	6.68E-03		L,D-carboxypeptidase A
CPE2580	3.73	5.60E-03		oxidoreductase, short chain dehydrogenase/reductase
CPE1190	3.73	1.37E-06		inner membrane transporter YjeM
CPE1255	3.73	5.28E-03	*hydG*	ferredoxin-NADP reductase
CPE1814	3.73	3.75E-03		stage IV sporulation protein B
CPE1956	3.72	7.15E-04		GntR family transcriptional regulator
CPE0429	3.71	1.15E-03		hypothetical protein

CPE2214	3.71	8.20E-04	*cphB*	cyanophycinase
CPE1800	3.70	4.09E-03		dihydroorotate dehydrogenase electron transfer subu
CPE1062	3.67	2.05E-03		electron transport complex protein RnfG
CPE1254	3.67	5.86E-03	*gltB*	oxidoreductase
CPE1649	3.65	1.18E-03		hypothetical protein
CPE0691	3.64	1.18E-03	*argG*	argininosuccinate synthase
CPE1437	3.64	1.25E-04	*hemA*	glutamyl-tRNA reductase
CPE0222	3.64	3.00E-03		sortase
CPE1978	3.60	3.38E-05	*asnA*	asparagine synthetase AsnA
CPE1381	3.58	1.05E-03		hypothetical protein
CPE2269	3.56	3.27E-06	*appF*	oligopeptide ABC transporter
CPE1600	3.55	5.18E-03	*thiH*	thiamine biosynthesis protein ThiH
CPE0221	3.54	7.71E-03		iron transporter
CPE1961	3.53	3.80E-04		sporulation protein YunB
CPE0600	3.53	3.26E-03		amino acid ABC transporter
CPE2545	3.52	3.05E-03		hypothetical protein
CPE0832	3.51	3.44E-03		hypothetical protein
CPE1180	3.49	1.01E-04	*pyrF*	orotidine 5'-phosphate decarboxylase
CPE0370	3.47	5.99E-04		transcriptional regulator
CPE1827	3.47	6.56E-03	*spoIIIAG*	stage III sporulation protein AG
CPE1065	3.46	8.49E-03		ferredoxin
CPE1660	3.45	1.08E-06	*feoB*	ferrous iron transport protein B
CPE1282	3.44	1.48E-03		transmembrane protein
CPE0186	3.44	1.39E-06		putative transporter
CPE2527	3.43	2.40E-03		hypothetical protein
CPE1409	3.41	3.62E-03		electron transport complex protein RnfC
CPE1579	3.41	2.14E-03		ABC transporter
CPE0190	3.41	1.50E-05		hypothetical protein
CPE0464	3.40	6.85E-03		spore coat polysaccharide biosynthesis protein
CPE2631	3.39	7.71E-03		PTS system protein
CPE1134	3.39	9.51E-03		hypothetical protein
CPE2273	3.39	2.87E-06	*dppA*	oligopeptide ABC transporter
CPE0940	3.39	1.99E-03		hypothetical protein
CPE0124	3.38	1.45E-03		hypothetical protein
PCP13	3.38	2.44E-03		hypothetical protein
CPE2533	3.38	6.21E-03	*spmA*	spore maturation protein A
CPE0557	3.35	7.31E-05		glycine betaine/carnitine/choline ABC transporter
CPE2608	3.34	2.03E-03		hypothetical protein
CPE0680	3.34	4.77E-04	*purL*	phosphoribosylformylglycinamidine synthase
CPE0235	3.33	1.91E-03		sensor histidine kinase
CPE0685	3.33	5.17E-04	*purN*	phosphoribosylglycinamide formyltransferase
CPE0945	3.32	1.16E-03		hypothetical protein
CPE2270	3.30	7.37E-06	*dppD*	oligopeptide ABC transporter
CPE1612	3.29	2.04E-03		hypothetical protein

CPE0891	3.29	2.80E-03		hypothetical protein
CPE0237	3.28	8.75E-03	*napA*	Na+/H+ antiporter
CPE1982	3.28	3.35E-03		hypothetical protein
CPE0684	3.28	2.36E-03	*purM*	phosphoribosylaminoimidazole synthetase
CPE1332	3.27	9.03E-03	*spoVR*	stage V sporulation protein R
CPE0220	3.27	6.77E-03		hypothetical protein
CPE0946	3.27	1.89E-03		hypothetical protein
CPE1781	3.27	2.48E-03		pheromone autoinducer 2 transporter
CPE0921	3.26	2.56E-03		nitroreductase
CPE1063	3.25	5.18E-03	*nqrD*	electron transport complex RsxE subunit
CPE1404	3.25	9.00E-03		hypothetical protein
CPE2183	3.25	3.75E-03	*spoIID*	stage II sporulation protein D
CPE1179	3.24	2.96E-04	*pyrZ*	dihydroorotate dehydrogenase electron transfer subunit
CPE1469	3.23	5.23E-03		aminotransferase class V
CPE0204	3.23	2.77E-03		acetyltransferase
CPE1383	3.22	3.35E-03		hypothetical protein
CPE1621	3.22	5.41E-05		hypothetical protein
CPE2045	3.20	4.46E-03	*spoVAE*	stage V sporulation protein AE
CPE1987	3.20	2.01E-03		sensor histidine kinase
CPE0665	3.19	1.93E-04		hypothetical protein
CPE1458	3.18	2.76E-03		hypothetical protein
CPE1202	3.17	4.93E-03		calcium-transporting ATPase
CPE2222	3.17	7.30E-03	*cotS*	spore coat protein
CPE0686	3.16	1.18E-03	*purH*	bifunctional phosphoribosylaminoimidazolecarboxamide formyltransferase/IMP cyclohydrolase
CPE0232	3.16	3.01E-03	*glpT*	glycerol-3-phosphate transporter
CPE2104	3.16	4.03E-03		hypothetical protein
CPE0849	3.16	4.14E-03		hypothetical protein
CPE0187	3.15	1.73E-06		cryptic beta-D-galactosidase subunit beta
CPE0404	3.15	1.63E-03	*brnQ*	branched chain amino acid ABC transporter carrier protein
CPE0050	3.15	3.75E-03		hypothetical protein
CPE0931	3.15	1.08E-04		coenzyme B12-dependent glycerol dehydrogenase small subunit
SR31	3.14	3.42E-06		Region between CPE0594 and CPE0595
CPE1896	3.13	2.94E-03		hypothetical protein
CPE2367	3.12	4.74E-03		N-acetylmuramoyl-L-alanine amidase
CPE2292	3.12	5.65E-03		hypothetical protein
CPE0950	3.12	4.16E-03		hypothetical protein
CPE2482	3.12	8.52E-03		stage V sprulation protein T
CPE0466	3.12	3.21E-03		hypothetical protein
CPE0738	3.11	1.77E-04		hypothetical protein
CPE2473	3.10	4.10E-03	*spoIIE*	stage II sprulation protein E
CPE2081	3.10	6.25E-05		ABC transporter
CPE0658	3.09	3.40E-03		hypothetical protein
CPE1802	3.08	3.33E-03		hypothetical protein

CPE0890	3.08	3.59E-03		hypothetical protein
CPE1025	3.08	7.84E-03	*pykA*	pyruvate kinase
CPE0456	3.06	3.56E-03		hypothetical protein
CPE0347	3.06	3.43E-03		transcriptional regulator
CPE1276	3.06	8.92E-03		multidrug efflux pump VmrA
CPE0867	3.03	4.57E-03		hypothetical protein
CPE1514	3.03	6.78E-04	*argI*	AlgI-related protein
CPE2054	3.03	5.84E-03		cation-transporting ATPase
CPE2559	3.02	4.74E-03		hypothetical protein
CPE0346	3.02	3.84E-03		transcriptional regulator
CPE2178	3.01	4.82E-03		hypothetical protein
CPE1238	3.01	1.01E-03		multidrug resistance protein
SR79	3.00	7.74E-06		Region between CPE2288 and CPE2289
CPE1230	3.00	4.03E-03		hypothetical protein
CPE2344	3.00	7.37E-06		carbohydrate diacid transcriptional activator CdaR
CPE0683	2.99	3.40E-03	*purF*	amidophosphoribosyltransferase
CPE2021	2.98	6.57E-03		sporulation protein YqfC
CPE1050	2.98	9.70E-03		5'-methylthioadenosine/S-adenosylhomocysteine nucleosidase
CPE0784	2.97	1.33E-03	*vgaB*	ABC transporter
CPE0245	2.96	3.44E-03		GGDEF/EAL domain protein
CPE2256	2.94	8.51E-04		oligopeptide-binding protein
CPE2246	2.94	2.83E-04		hypothetical protein
CPE0386	2.93	3.78E-04		transcriptional regulator
CPE0644	2.93	8.66E-06	*flaV*	flavodoxin
CPE1122	2.91	9.00E-03		phage-related head protein
CPE1143	2.91	4.65E-03		hypothetical protein
CPE1139	2.90	9.11E-03		hypothetical protein
CPE1212	2.89	3.43E-05	*uraA*	uracil permease
CPE2585	2.89	5.37E-03		nucleoside recognition domain protein
CPE1271	2.87	3.67E-03		ABC transporter
CPE1324	2.86	9.15E-03		amino acid ABC transporter
CPE0564	2.86	4.21E-03	*spoVD*	stage V sporulation protein D
CPE1960	2.85	2.13E-05	*hprT*	hypoxanthine phosphoribosyltransferase
SR13	2.84	3.50E-03		Region between CPE0143 and CPE0144
CPE1565	2.82	3.92E-04		putative calcium/sodium:proton antiporter
CPE1379	2.81	6.24E-03		D-alanyl-D-alanine carboxypeptidase
CPE0383	2.81	7.43E-03		hypothetical protein
CPE1522	2.80	5.91E-03		hypothetical protein
CPE0814	2.80	7.93E-03		GNAT family acetyltransferase
CPE0762	2.77	7.17E-04		HAD superfamily (subfamily IA) hydrolase
CPE1287	2.76	7.60E-03		hypothetical protein
CPE0161	2.75	7.32E-03		hypothetical protein
CPE1018	2.75	8.34E-03		4-amino-4-deoxychorismate lyase
CPE2271	2.75	9.61E-05	*dppC*	oligopeptide ABC transporter

CPE0092	2.74	7.32E-03		symporter YidK
CPE0336	2.74	6.91E-03		threonine and homoserine efflux system
CPE0431	2.73	6.16E-03		hypothetical protein
CPE0283	2.72	3.88E-05		hypothetical protein
CPE0335	2.72	5.71E-04	*xpt*	xanthine phosphoribosyltransferase
CPE0453	2.70	8.22E-03		MerR family transcriptional regulator
CPE0792	2.70	4.65E-04		iron(III) dicitrate ABC transporter
CPE1828	2.69	8.64E-03		stage III sporulation protein AF
CPE1477	2.69	9.92E-03		polysaccharide deacetylase
CPE2121	2.68	1.95E-04	*dacF*	D-alanyl-D-alanine carboxypeptidase
CPE2107	2.67	3.37E-03		hypothetical protein
CPE2453	2.67	1.63E-03		Orn/Lys/Arg decarboxylase
CPE2607	2.67	6.17E-04		hypothetical protein
CPE0757	2.67	4.37E-03		creatinase
CPE1046	2.66	7.25E-03		putative alpha-glucosidase
CPE1343	2.65	1.36E-03	*mglC*	beta-methylgalactoside transporter inner membrane component
CPE1365	2.65	7.26E-04		hypothetical protein
CPE0219	2.63	9.96E-03		AraC family transcriptional regulator
SR41	2.62	1.61E-04		Region between CPE0826 and CPE0827
CPE2272	2.61	8.65E-05	*dppB*	oligopeptide ABC transporter
CPE0930	2.60	2.18E-04		coenzyme B12-dependent glycerol dehydrogenase medium subunit
CPE1293	2.60	7.76E-03		tetratricopeptide repeat protein
PCP30	2.59	7.25E-03		hypothetical protein
CPE1507	2.57	8.34E-05		endo-1,4-beta-xylanase
CPE1913	2.56	4.41E-05		pheromone autoinducer 2 transporter
CPE1659	2.56	3.35E-05	*feoA*	ferrous iron transport protein A
CPE0481	2.56	7.27E-03		beta-1,4-galactosyltransferase
CPE0520	2.54	1.43E-04		hypothetical protein
CPE2280	2.53	9.20E-03		hypothetical protein
CPE1912	2.52	2.13E-04		hypothetical protein
CPE0558	2.52	9.50E-05		glycine betaine/carnitine/choline ABC transporter
CPE0759	2.51	6.95E-04		carbohydrate diacid transcriptional activator CdaR
CPE0663	2.51	7.31E-05		F5/8 type C domain protein
CPE0632	2.51	1.06E-04	*brnQ*	branched chain amino acid ABC transporter carrier protein
CPE1415	2.51	2.87E-05		alpha-mannosidase
CPE1247	2.50	1.29E-03	*mprF2*	lysyl-tRNA synthetase
CPE0021	2.46	1.19E-05	*addA*	ATP-dependent deoxyribonuclease chain A
CPE0681	2.46	7.49E-03	*purE*	phosphoribosylaminoimidazole carboxylase, catalytic subunit
CPE0508	2.45	6.86E-03		hypothetical protein
CPE2617	2.43	2.87E-03		acyl-ACP thioesterase
CPE0828	2.43	1.00E-03		hypothetical protein
CPE2505	2.41	9.03E-03		MATE efflux family protein
CPE0754	2.41	1.73E-05	*clsC*	cardiolipin synthetase

CPE0430	2.40	1.74E-03	*sipS*	type I signal peptidase
SR78	2.39	9.46E-04		Region between CPE2276 and CPE2277
CPE1396	2.39	1.74E-04		ATP-dependent RNA helicase, DEAD/DEAH box family
CPE1481	2.36	9.05E-05		hypothetical protein
CPE1505	2.36	2.28E-05	*uraA*	uracil transporter
CPE2010	2.36	7.39E-03	*cotS*	spore coat protein
PCP63	2.36	5.69E-05		hypothetical protein
SR39	2.36	8.65E-04		Region between CPE0738 and CPE0739
CPE1016	2.31	5.03E-03	*pabA*	anthranilate synthase component II
CPE0254	2.31	4.65E-03		hypothetical protein
CPE1979	2.31	1.71E-03		hypothetical protein
SR90	2.31	1.86E-04		Region between CPE2541 and CPE2542
SR37	2.29	9.08E-05		Region between CPE0709 and CPE0710
CPE1529	2.29	3.10E-04		hypothetical protein
CPE1241	2.26	5.07E-04		hypothetical protein
CPE1926	2.25	3.24E-04		sensor histidine kinase
CPE1426	2.24	6.70E-04		hypothetical protein
CPE0028	2.24	1.56E-03	*psdD*	phosphatidylserine decarboxylase
CPE0676	2.23	7.08E-03		hypothetical protein
CPE2560	2.23	5.65E-03		spore cortex-lytic enzyme SleC
CPE0072	2.23	3.84E-03		putative aminobenzoyl-glutamate transporter
CPE1598	2.23	1.77E-04		hypothetical protein
CPE0929	2.22	1.28E-04		coenzyme B12-dependent glycerol dehydrogenase large subunit
CPE1026	2.21	7.95E-03		undecaprenyl pyrophosphate phosphatase
CPE0263	2.20	1.77E-04		hypothetical protein
CPE1520	2.20	5.52E-03	*ilvE*	branched-chain amino acid aminotransferase
CPE0074	2.19	2.03E-03		transcription antiterminator
CPE1476	2.18	3.12E-04		hypothetical protein
CPE1161	2.18	8.65E-05	*lexA*	LexA repressor
CPE1556	2.17	2.55E-03		hypothetical protein
CPE1284	2.17	3.77E-03		pyrimidine nucleoside transporter
CPE0084	2.16	1.38E-04	*iolR*	transcription repressor of myo-inositol catabolism operon
CPE0932	2.16	3.59E-04		glycerol dehydratase reactivation factor, large subunit
CPE2648	2.15	6.27E-03		sporulation integral membrane protein YtvI
SR89	2.14	1.88E-04		Region between CPE2527 and CPE2528
CPE0725	2.14	3.83E-05	*nanI*	exo-alpha-sialidase
CPE0472	2.14	3.35E-03		ABC transporter
CPE0836	2.14	8.88E-03		hypothetical protein
CPE0736	2.13	4.18E-03		amino acid ABC transporter permease
CPE2080	2.13	4.64E-04		alpha-mannosidase
CPE0714	2.13	1.14E-04		diacylglycerol glucosyltransferase
CPE1163	2.12	2.26E-04	*xerC*	site-specific tyrosine recombinase XerC
CPE1144	2.10	6.07E-04		transcriptional regulator
CPE2358	2.08	1.65E-04		sigma-L-dependent transcriptional regulator

CPE1500	2.08	8.73E-03	*virS*	sensor histidine kinase VirS
CPE1051	2.07	5.89E-04	*cfa*	cyclopropane-fatty-acyl-phospholipid synthase
CPE1751	2.05	4.70E-03		hypothetical protein
CPE0207	2.01	4.47E-03		two-component sensor histidine kinase
CPE2103	2.01	3.42E-03		ribosomal protein L11 methyltransferase
PCP59	2.01	1.89E-04		hypothetical protein
CPE0978	2.00	4.17E-03		potassium transport protein Kup
CPE2553	1.99	1.52E-03	*glpP*	glycerol uptake operon antiterminator
CPE1456	1.98	9.00E-03		ABC transporter
CPE0827	1.98	9.61E-05		polysaccharide deacetylase
CPE1283	1.98	2.14E-03		transcriptional regulator
CPE1374	1.95	1.48E-03		calcium/proton exchanger
CPE1882	1.94	6.39E-04		peptidase U32
CPE2053	1.94	1.49E-03		hypothetical protein
CPE1563	1.91	9.64E-04		hypothetical protein
CPE2247	1.91	8.02E-03		2-aminoethylphosphonate ABC transporter substrate-b
CPE1787	1.90	5.36E-03		recombination factor protein RarA
PCP60	1.90	1.66E-04		hypothetical protein
CPE0949	1.90	8.29E-03		ABC transporter
PCP62	1.90	2.80E-03		hypothetical protein
CPE1623	1.89	1.03E-03		lipoprotein
CPE0933	1.89	4.11E-03		glycerol dehydratase reactivation factor, small subunit
CPE0289	1.89	3.39E-04		endo-beta-N-acetylglucosaminidase
CPE0999	1.89	1.25E-04		hypothetical protein
CPE0073	1.88	6.24E-03		transcription antiterminator
CPE2159	1.88	9.18E-04		chromosome segregation protein
SR43	1.88	1.90E-03		Region between CPE0878 and CPE0879
CPE0437	1.87	2.57E-04	*hlyB*	hemolysin
CPE1457	1.87	3.50E-03		ABC transporter
SR51	1.87	8.74E-05		Region between CPE1166 and CPE1167
CPE1228	1.86	2.35E-03	*cbiK*	CbiK protein
CPE0426	1.86	9.96E-04		hypothetical protein
CPE2078	1.86	1.96E-03		hypothetical protein
CPE0841	1.86	8.87E-03		two-component sensor histidine kinase
CPE0104	1.85	1.57E-03		hypothetical protein
CPE0071	1.84	6.51E-03	*clsA*	cardiolipin synthase
CPE1864	1.84	2.21E-04		hypothetical protein
CPE2498	1.82	1.82E-03		Ser/Thr protein phosphatase
CPE2296	1.82	4.78E-03		ABC transporter
PCP58	1.81	6.17E-04		hypothetical protein
CPE1976	1.81	1.33E-03	*dacF*	D-alanyl-D-alanine carboxypeptidase
SR71	1.81	6.64E-03		Region between CPE2071 and CPE2072
CPE1551	1.80	3.39E-04		endo-1,4-beta-xylanase
CPE1658	1.79	5.81E-03		hypothetical protein

CPE0035	1.79	2.34E-04	*yplC*	hypothetical protein
CPE0772	1.78	5.22E-03		hypothetical protein
CPE0646	1.77	4.16E-04		hypothetical protein
CPE1727	1.75	3.50E-03		hypothetical protein
CPE0970	1.75	2.43E-03		Na+/H+ antiporter
CPE1880	1.73	2.62E-03	*spoVD*	stage V sporulation protein D
CPE0162	1.73	3.81E-03	*pfoR*	regulatory protein
CPE2522	1.71	8.77E-03		cyclic nucleotide-binding protein
CPE2094	1.70	4.67E-03		NADH-plastoquinone oxidoreductase subunit
CPE2494	1.69	4.77E-04		transcriptional regulator
CPE1759	1.68	9.37E-04	*nrdR*	transcriptional regulator NrdR
CPE0434	1.68	9.72E-04		GNAT family acetyltransferase
CPE2592	1.65	1.10E-03	*phnA*	phnA family protein
CPE0595	1.64	5.36E-03	*lysA*	diaminopimelate decarboxylase
CPE2165	1.64	1.45E-03		glycoprotease
CPE1316	1.64	3.21E-03		two-component sensor histidine kinase
CPE2082	1.63	4.37E-03		ABC transporter
CPE1767	1.63	4.77E-04	*pilT*	twitching motility protein PilT
CPE1169	1.62	6.95E-04		hypothetical protein
CPE0184	1.61	1.10E-03	*nanP*	N-acetylmannosamine-6-phosphate 2-epimerase
CPE0004	1.60	7.39E-04	*recF*	recombination protein F
CPE1779	1.60	4.14E-03		hypothetical protein
CPE1166	1.59	1.22E-03		lysine specific permease
SR5	1.57	2.75E-03		Region between CPE0075 and CPE0076
CPE0185	1.57	1.07E-03	*nanA*	N-acetylneuraminate lyase
CPE2216	1.56	1.58E-03		hypothetical protein
CPE2475	1.56	1.93E-03		hypothetical protein
CPE2590	1.54	4.10E-03		phage infection protein
CPE0789	1.54	1.97E-03		transcriptional regulator
CPE0671	1.53	7.18E-03		hypothetical protein
CPE1914	1.53	1.94E-03		PBP 5 synthesis repressor
CPE1847	1.53	5.16E-03		fibronectin-binding protein
CPE0291	1.53	3.04E-03	*acpS*	4'-phosphopantetheinyl transferase
CPE1653	1.51	3.67E-03		hypothetical protein
CPE0314	1.50	2.05E-03		hypothetical protein
CPE2307	1.49	8.87E-03		hypothetical protein
CPE0882	1.49	1.85E-03		hypothetical protein
CPE1963	1.47	1.64E-03		hypothetical protein
CPE0782	1.47	7.32E-03		alkyl hydroperoxide reductase
CPE1430	1.47	5.22E-03	*clsD*	cardiolipin synthase
PCP33	1.46	2.27E-03		hypothetical protein
CPE2206	1.46	6.64E-03		monogalactosyldiacylglycerol synthase
CPE1329	1.43	1.04E-03		CDP-diacylglycerol--serine O-phosphatidyltransferase
CPE1445	1.42	1.91E-03		AraC family transcriptional regulator

CPE1227	1.42	8.49E-03	*cbiC*	precorrin-8X methylmutase
CPE0953	1.42	5.36E-03		pyridine nucleotide-disulphide oxidoreductase
CPE1825	1.40	1.35E-03		alkaline shock protein
CPE0766	1.40	3.31E-03		hypothetical protein
CPE2531	1.40	1.94E-03	*adhE*	bifunctional acetaldehyde-CoA/alcohol dehydrogenase
CPE0610	1.40	1.51E-03		chromosome segregation protein
CPE1634	1.39	1.19E-03	*tagO*	undecaprenyl-phosphate N-acetylglucosaminyltransferase
CPE1346	1.39	1.08E-03	*galT*	galactose-1-phosphate uridylyltransferase
CPE0180	1.39	7.65E-03		hypothetical protein
CPE0605	1.38	1.45E-03		cation efflux family protein
CPE1171	1.36	3.62E-03		hypothetical protein
CPE1883	1.35	8.96E-03		histidinol-phosphatase
CPE0662	1.35	3.37E-03		photosystem I assembly protein Ycf3
CPE1203	1.33	6.27E-03	*spoVB*	stage V sporulation protein B
CPE1210	1.32	8.39E-03		FUR family transcriptional regulator
CPE2012	1.32	3.54E-03		CBS domain protein
CPE2145	1.32	2.02E-03	*maf*	Maf-like protein
CPE1664	1.31	3.05E-03		peptidase, M50 family
CPE0159	1.31	8.69E-03		hypothetical protein
CPE1506	1.31	2.99E-03		ABC transporter
CPE2539	1.30	4.96E-03	*ccpA*	transcriptional regulator
CPE2474	1.30	2.57E-03		general stress protein 13
CPE2345	1.30	2.32E-03		maltose ABC transportor
CPE2275	1.29	4.87E-03	*guaA*	GMP synthase
CPE0136	1.28	5.52E-03		hypothetical protein
CPE0100	1.28	6.72E-03		dihydroxyacetone kinase
CPE0922	1.27	3.85E-03		hypothetical protein
CPE0005	1.26	4.60E-03		hypothetical protein
CPE1475	1.25	3.93E-03		LysR family transcriptional regulator
CPE2237	1.25	2.98E-03		arylsulfatase
SR94	1.24	7.50E-03		Region between CPE1138 and CPE1139
PCP32	1.23	5.95E-03		resolvase
CPE2158	1.21	6.90E-03		ABC transporter substrate-binding protein
CPE1015	1.21	6.49E-03		error-prone DNA polymerase
CPE1153	1.20	8.19E-03	*pflA*	formate acetyltransferase
CPE2548	1.20	6.88E-03		methyltransferase
CPE0452	1.20	3.93E-03	*entC*	enterotoxin
CPE1281	1.19	4.27E-03		hypothetical protein
CPE1701	1.19	6.68E-03		transcriptional repressor CodY
CPE0856	1.17	5.99E-03		alpha-mannosidase
CPE1167	1.16	7.93E-03		glutamate/ aspartate transporter
CPE0189	1.15	4.52E-03		RpiR family transcriptional regulator
SR69	1.15	7.13E-03		Region between CPE1977 and CPE1978
CPE1540	1.12	8.18E-03		ABC transporter

CPE1191	1.11	9.82E-03		hypothetical protein
CPE0310	1.03	9.44E-03		L-lactate permease
CPE0937	-1.02	9.11E-03		amino acid permease
CPE0791	-1.04	7.32E-03		NAD(P)H dehydrogenase
CPE0904	-1.06	8.67E-03	*eutT*	ethanolamine utilization cobalamin adenosyltransferase
CPE1741	-1.06	5.56E-03		RNA-binding Sun protein
CPE2425	-1.06	7.25E-03	*thyX*	FAD-dependent thymidylate synthase
CPE1344	-1.06	7.41E-03		aldose 1-epimerase
CPE2138	-1.07	7.13E-03	*minD*	septum site-determining protein MinD
CPE1009	-1.07	6.41E-03	*mgsA*	methylglyoxal synthase
CPE1906	-1.07	4.94E-03	*dapB*	dihydrodipicolinate reductase
CPE1746	-1.09	4.47E-03		bifunctional phosphopantothenoylcysteine decarboxylase/phosphopantothenate synthase
CPE1196	-1.09	6.12E-03		hypothetical protein
CPE1573	-1.09	8.59E-03		bifunctional glutamate--cysteine ligase/glutathione synthetase
CPE2335	-1.09	9.92E-03		hypothetical protein
CPE0046	-1.09	7.76E-03		hypothetical protein
CPE2459	-1.09	7.16E-03	*murD*	UDP-N-acetylmuramoyl-L-alanyl-D-glutamate synthetase
CPE0806	-1.09	7.47E-03		hypothetical protein
SR48	-1.09	8.01E-03		Region between CPE0997 and CPE0998
CPE0625	-1.10	6.43E-03		hypothetical protein
CPE2197	-1.10	8.07E-03	*upp*	uracil phosphoribosyltransferase
PCP57	-1.10	8.22E-03	*cna*	collagen adhesin
CPE0031	-1.10	7.82E-03		cytidine/deoxycytidylate deaminase
CPE1683	-1.11	5.49E-03	*truB*	tRNA pseudouridine synthase B
CPE0510	-1.12	3.86E-03	*galF*	UTP-glucose-1-phosphate uridylyltransferase
CPE2133	-1.13	9.20E-03		radical SAM domain protein
CPE2015	-1.13	5.65E-03	*era*	GTP-binding protein Era
CPE0982	-1.13	8.79E-03		hypothetical protein
CPE2259	-1.14	3.73E-03	*ligA*	NAD-dependent DNA ligase LigA
CPE0008	-1.14	2.71E-03		DNA gyrase subunit A
CPE1734	-1.16	4.47E-03	*rpmB*	50S ribosomal protein L28
CPE2659	-1.16	2.63E-03	*rnpA*	ribonuclease P
CPE0056	-1.17	4.46E-03	*mtn*	5'-methylthioadenosine/S-adenosylhomocysteine nucleosidase
CPE1755	-1.17	3.45E-03	*engA*	GTP-binding protein EngA
CPE2170	-1.17	3.54E-03	*prfB*	peptide chain release factor 2
CPE1859	-1.18	2.37E-03	*spoVE*	stage V sporulation protein E
CPE1503	-1.18	7.26E-03		hypothetical protein
CPE2202	-1.19	5.36E-03	*prfA*	peptide chain release factor 1
CPE2657	-1.20	1.93E-03	*spoIIIJ*	inner membrane protein translocase component YidC
CPE1187	-1.20	3.75E-03	*manA*	mannose-6-phosphate isomerase
CPE1234	-1.20	5.47E-03	*nagJ*	hyaluronidase
CPE2605	-1.20	6.77E-03		metallo-beta-lactamase

CPE0300	-1.20	3.21E-03	*ftsX*	cell-division protein
CPE0626	-1.21	7.47E-03		hypothetical protein
PCP51	-1.21	6.70E-03		type IV secretion system protein VirD4
CPE0742	-1.22	3.06E-03	*lipB*	lipase
CPE0823	-1.23	2.80E-03		PTS system
CPE1971	-1.23	4.52E-03	*potD*	spermidine/putrescine ABC transporter
CPE0353	-1.23	4.59E-03	*murB*	UDP-N-acetylenolpyruvoylglucosamine reductase
CPE0375	-1.23	5.37E-03		endo-beta-galactosidase C
CPE0549	-1.23	3.75E-03	*dclY*	Orn/Lys/Arg decarboxylase
CPE1946	-1.23	6.16E-03	*queA*	S-adenosylmethionine:tRNA ribosyltransferase-isomerase
CPE0110	-1.24	4.34E-03	*thiH*	thiamine biosynthesis protein ThiH
CPE0150	-1.24	5.99E-03		2-dehydro-3-deoxyphosphogluconate aldolase/4-hydroxy-2-oxoglutarate aldolase
CPE0216	-1.24	4.01E-03	*sbcC*	exonuclease SbcC
CPE2355	-1.25	4.42E-03		thioredoxin
CPE1389	-1.25	2.27E-03	*engB*	ribosome biogenesis GTP-binding protein YsxC
CPE0032	-1.25	7.76E-03		hypothetical protein
CPE0299	-1.26	2.32E-03	*ftsE*	cell-division ATP-binding protein
CPE0305	-1.26	5.65E-03		ABC transporter
CPE2495	-1.27	5.97E-03	*punA*	purine nucleoside phosphorylase
CPE2418	-1.27	3.78E-03	*nusG*	transcription antitermination protein NusG
PCP50	-1.27	4.56E-03		hypothetical protein
CPE2339	-1.28	1.47E-03		alpha-glucosidase
CPE2168	-1.28	1.91E-03		30S ribosomal protein S1
CPE2004	-1.28	2.21E-03		metal-binding protein
CPE0989	-1.29	2.99E-03	*sdhA*	L-serine dehydratase alpha subunit
CPE2507	-1.29	2.40E-03	*nrdD*	anaerobic ribonucleoside triphosphate reductase
CPE2196	-1.30	2.48E-03		UDP-N-acetylglucosamine 2-epimerase
CPE0447	-1.30	1.51E-03	*lguL*	lactoylglutathione lyase
CPE2322	-1.31	5.01E-03	*thrS*	threonyl-tRNA synthetase
CPE2357	-1.31	2.81E-03		phosphoenolpyruvate-protein phosphotransferase
CPE1314	-1.31	4.93E-03		muramidase
CPE1740	-1.31	9.96E-04		ribosomal RNA large subunit methyltransferase N
CPE2419	-1.32	2.75E-03	*secE*	preprotein translocase subunit SecE
CPE1682	-1.32	2.20E-03	*ribC*	bifunctional riboflavin kinase/FMN adenylyltransferase
CPE0577	-1.32	6.90E-03		ABC transporter permease
CPE2193	-1.32	3.24E-03	*atpI*	ATP synthase F0F1 subunit A
CPE1860	-1.33	1.80E-03	*mraY*	phospho-N-acetylmuramoyl-pentapeptide- transferase
CPE1690	-1.33	1.88E-03		ribosome maturation protein RimP
CPE0741	-1.33	3.26E-03		cytochrome C-type biogenesis protein
CPE2464	-1.33	1.26E-03	*glyS*	glycyl-tRNA synthetase
CPE2332	-1.34	3.33E-03		two-component response regulator
CPE1392	-1.34	2.78E-03	*clpX*	ATP-dependent protease ATP-binding subunit ClpX
CPE0393	-1.34	1.89E-03		tartronate semialdehyde reductase
CPE1945	-1.34	1.48E-03	*tgt*	queuine tRNA-ribosyltransferase

CPE1628	-1.34	3.31E-03		ribose ABC transporter
CPE1236	-1.34	1.59E-03	*sod*	Mn/Fe superoxide dismutase
CPE0506	-1.35	1.86E-03	*galF*	UTP-glucose-1-phosphate uridylyltransferase
CPE0654	-1.35	1.39E-03	*relA*	relA/spoT family protein
CPE2027	-1.36	2.85E-03	*prmA*	ribosomal protein L11 methyltransferase
CPE0323	-1.36	1.81E-03		PTS system protein
CPE2603	-1.36	2.63E-03		preprotein translocase subunit SecA
CPE2208	-1.36	1.29E-03	*pyrG*	CTP synthetase
CPE0199	-1.36	1.29E-03	*malH*	maltose-6'-phosphate glucosidase
CPE1748	-1.36	1.23E-03	*gmk*	guanylate kinase
CPE0604	-1.37	8.76E-04		arylsulfatase
CPE0301	-1.37	2.35E-03		carboxyl-terminal protease
CPE1525	-1.37	6.68E-03		ABC transporter
PCP29	-1.37	2.26E-03		hypothetical protein
CPE2624	-1.38	1.49E-03	*sdhA*	L-serine dehydratase alpha subunit
CPE0743	-1.38	1.68E-03	*cstA*	carbon starvation protein CstA
CPE0389	-1.38	3.20E-03	*arcD*	amino acid permease
CPE2406	-1.39	7.15E-04	*rpsJ*	30S ribosomal protein S10
CPE1508	-1.39	2.68E-03	*topB*	DNA topoisomerase III
CPE0012	-1.40	1.10E-03		hydroxyglutarate oxidase
CPE2432	-1.40	1.09E-03		DNA integrity scanning protein DisA
CPE2163	-1.40	2.99E-03		CBS domain protein
CPE1315	-1.40	1.91E-03	*ddl*	D-alanine--D-alanine ligase
CPE2293	-1.41	9.64E-04		8-oxoguanine DNA glycosylase
CPE2653	-1.41	5.03E-03	*gidB*	16S rRNA methyltransferase GidB
CPE0804	-1.41	9.80E-04		sensory box protein/histidinol phosphate phosphatase
CPE1743	-1.42	1.10E-03	*fmt*	methionyl-tRNA formyltransferase
CPE0633	-1.42	2.56E-03	*trpS*	tryptophanyl-tRNA synthetase
CPE1323	-1.42	2.78E-03		CBS domain/MgtE intracellular domain protein
CPE1890	-1.42	1.11E-03	*trkA*	potassium uptake system protein
CPE1933	-1.43	7.87E-04	*aspS*	aspartyl-tRNA synthetase
CPE0928	-1.43	2.28E-03		two-component response regulator
CPE2380	-1.44	1.49E-03	*rpmJ*	50S ribosomal protein L36
CPE1936	-1.44	9.64E-04		metallo-beta-lactamase
CPE1382	-1.44	1.10E-03	*map*	methionine aminopeptidase, type I
CPE0047	-1.44	7.50E-03	*recR*	recombination protein RecR
CPE0002	-1.45	6.95E-04	*dnaN*	DNA polymerase III subunit beta
CPE2150	-1.45	1.80E-03	*rpsA*	30S ribosomal protein S1
CPE1715	-1.45	1.33E-03	*ftsY*	signal recognition particle-docking protein FtsY
CPE1670	-1.46	6.25E-04	*aspC*	aspartate aminotransferase
CPE0599	-1.46	7.17E-04		DNA-dependent helicase II
CPE2636	-1.46	1.91E-03	*rplI*	50S ribosomal protein L9
CPE0988	-1.47	5.73E-03	*sdhB*	L-serine dehydratase beta subunit
CPE1142	-1.47	1.33E-03		hypothetical protein

CPE0422	-1.47	2.87E-03		L-fucose transporter
CPE0551	-1.47	5.62E-04	*speB*	agmatinase
SR81	-1.48	1.42E-03		Region between CPE2302 and CPE2303
CPE1554	-1.48	1.17E-03		lipoprotein
CPE2059	-1.49	1.99E-03		hypothetical protein
CPE1013	-1.49	1.69E-03		hypothetical protein
CPE0361	-1.49	6.39E-04	*pfkA*	6-phosphofructokinase
CPE1630	-1.50	1.29E-03	*rbsA*	ribose ABC transporter
CPE1492	-1.50	5.35E-03		hypothetical protein
CPE0304	-1.50	6.07E-04		degV family protein
CPE0981	-1.51	4.69E-03		glycosyl hydrolase, family 20
CPE2640	-1.51	1.18E-03	*rpsR*	30S ribosomal protein S18
CPE0689	-1.51	2.08E-03		hypothetical protein
CPE1944	-1.51	5.56E-04	*yajC*	preprotein translocase subunit YajC
CPE1629	-1.52	1.00E-03	*rbsC*	ribose ABC transporter
CPE1232	-1.52	9.19E-04		hypothetical protein
CPE2412	-1.52	1.18E-03	*rpoC*	DNA-directed RNA polymerase subunit beta'
CPE0656	-1.53	1.24E-03		hypothetical protein
CPE2656	-1.53	3.92E-04	*jag*	SpoIIIJ-associated protein
CPE1463	-1.53	9.00E-04		PTS system protein
CPE1892	-1.54	4.96E-04	*rplT*	50S ribosomal protein L20
CPE1894	-1.54	7.60E-04	*infC*	translation initiation factor IF-3
CPE2524	-1.54	4.50E-04	*metG*	methionyl-tRNA synthetase
CPE0013	-1.55	6.48E-03		hypothetical protein
CPE1934	-1.55	5.78E-04	*hisS*	histidyl-tRNA synthetase
CPE1390	-1.56	5.02E-04	*lonA*	ATP-dependent protease La
CPE2023	-1.56	7.17E-04	*rpsU*	30S ribosomal protein S21
CPE1744	-1.57	1.12E-03	*def*	peptide deformylase
CPE1631	-1.57	5.11E-04	*rbsD*	D-ribose pyranase
CPE0079	-1.57	8.83E-04	*fus*	elongation factor G
CPE1453	-1.57	6.29E-04	*pepF*	oligoendopeptidase F
CPE2350	-1.57	6.95E-04		hypothetical protein
CPE0322	-1.58	1.16E-03		PTS system protein
CPE2062	-1.58	7.33E-04		flavodoxin
CPE1279	-1.58	4.97E-04	*nagK*	hyaluronidase
CPE1835	-1.59	4.70E-04	*efp*	elongation factor P
CPE0328	-1.59	8.98E-04	*lacC*	tagatose-6-phosphate kinase
CPE2330	-1.60	7.83E-04		hypothetical protein
CPE1754	-1.60	4.50E-04	*gpsA*	NAD(P)H-dependent glycerol-3-phosphate dehydrogenase
CPE1076	-1.60	8.93E-03	*accA*	acetyl-CoA carboxylase carboxyltransferase subunit alpha
CPE2381	-1.61	5.02E-04	*infA*	translation initiation factor IF-1
CPE1452	-1.61	4.76E-04		rhomboid family protein
CPE1943	-1.61	3.23E-04		astB/chuR-related protein
CPE2602	-1.62	8.76E-04		hypothetical protein

CPE2118	-1.62	3.89E-03	*selD*	selenophosphate synthetase
CPE2176	-1.62	4.76E-04	*nagA*	N-acetylglucosamine-6-phosphate deacetylase
CPE1393	-1.62	5.17E-04	*clpP*	ATP-dependent Clp protease proteolytic subunit
CPE0858	-1.63	1.32E-03	*bdhA*	NADH-dependent butanol dehydrogenase
CPE1295	-1.64	4.81E-04	*smpB*	SsrA-binding protein
CPE2177	-1.64	5.56E-04	*metK*	S-adenosylmethionine synthetase
CPE1845	-1.64	9.84E-03		hypothetical protein
CPE0822	-1.64	5.25E-04		PTS system, mannose/fructose/sorbose family, IIC component
CPE1784	-1.66	9.95E-04	*nifU*	nitrogen fixation protein
CPE0354	-1.66	2.46E-04		glmZ(sRNA)-inactivating NTPase
CPE2326	-1.66	2.42E-03		hypothetical protein
CPE0132	-1.66	4.17E-04	*cinA*	competence damage-inducible protein A
CPE1004	-1.67	3.10E-04	*hprK*	HPr kinase/phosphorylase
CPE0533	-1.68	9.00E-03		hypothetical protein
CPE0313	-1.69	1.81E-03		glycolate oxidase subunit
CPE1885	-1.70	1.97E-04	*pheT*	phenylalanyl-tRNA synthetase subunit beta
CPE1886	-1.70	6.91E-04	*pheS*	phenylalanyl-tRNA synthetase subunit alpha
CPE1434	-1.70	3.37E-03	*hemX*	uroporphyrinogen-III methyltransferase/synthase
PCP39	-1.70	3.21E-03		hypothetical protein
CPE0165	-1.70	1.18E-03		hypothetical protein
CPE0995	-1.71	4.03E-04		glucanase
CPE0471	-1.71	7.60E-04		hypothetical protein
CPE0320	-1.72	1.89E-03		PTS system mannose-specific transporter subunits II
CPE1708	-1.72	2.26E-04	*rplS*	50S ribosomal protein L19
CPE2210	-1.72	7.81E-03		thiamine biosynthesis protein ThiI
CPE0494	-1.73	1.62E-03		NDP-suger dehydrogenase
CPE2552	-1.73	1.74E-04	*glpK*	glycerol kinase
CPE1736	-1.73	6.95E-04		ribulose-phosphate 3-epimerase
CPE0916	-1.73	9.95E-04		hypothetical protein
CPE0280	-1.73	2.99E-03		tetrapyrrole methylase
CPE2071	-1.73	4.96E-04		glycosyl transferase, group 2 family protein
CPE1632	-1.73	4.77E-04	*rbsK*	ribokinase
CPE1846	-1.74	2.60E-03	*dapF*	diaminopimelate epimerase
CPE1747	-1.75	2.72E-04	*rpoZ*	DNA-directed RNA polymerase subunit omega
CPE0173	-1.75	3.10E-04	*colA*	collagenase
CPE1919	-1.75	2.32E-04	*valS*	valyl-tRNA synthetase
CPE0081	-1.76	1.02E-03	*glcK*	glucose kinase
CPE2413	-1.76	1.97E-04	*rpoB*	DNA-directed RNA polymerase subunit beta
CPE0411	-1.76	1.89E-03		monooxygenase
CPE1866	-1.76	2.15E-04		GTP-dependent nucleic acid-binding protein EngD
CPE2427	-1.77	3.86E-04	*cysS*	cysteinyl-tRNA synthetase
CPE2022	-1.77	2.59E-04		GatB/Yqey domain protein
CPE2337	-1.77	2.13E-04	*glgP*	glycogen phosphorylase
CPE1504	-1.78	2.32E-03		hypothetical protein

CPE0550	-1.78	2.55E-04	*speE*	spermidine synthase
CPE2401	-1.78	3.65E-04	*rpsS*	30S ribosomal protein S19
CPE2567	-1.79	2.18E-04		hypothetical protein
CPE2167	-1.79	1.09E-04		hypothetical protein
CPE0591	-1.79	1.16E-03		glutaminase
CPE1733	-1.80	3.98E-04		hypothetical protein
CPE2143	-1.81	9.08E-05	*mreB*	rod shape-determining protein MreB
SR18	-1.81	2.13E-04	*trnA-met*	Region between CPE0238 and CPE0239
CPE0003	-1.81	7.93E-03		S4 domain protein
CPE2067	-1.82	2.16E-04		DJ-1 family protein
SR85	-1.82	9.61E-05		Region between CPE2406 and CPE2407
CPE1092	-1.82	2.13E-04	*pac*	choloylglycine hydrolase
CPE1893	-1.83	1.94E-04	*rpmI*	50S ribosomal protein L35
CPE1686	-1.83	1.95E-04	*infB*	translation initiation factor IF-2
CPE0715	-1.83	6.89E-04		GNAT family acetyltransferase
CPE0102	-1.84	5.92E-04		hypothetical protein
CPE1432	-1.84	5.15E-04	*hemL*	glutamate-1-semialdehyde aminotransferase
CPE1045	-1.84	7.15E-04	*cbiP*	cobyric acid synthase
CPE1719	-1.84	5.56E-04	*acpP*	acyl carrier protein
SR54	-1.86	5.17E-04		Region between CPE1233 and CPE1234
CPE2470	-1.86	2.89E-04	*ftsH*	ATP-dependent metalloprotease FtsH
CPE1642	-1.86	1.80E-04	*ntpK*	V-type ATP synthase subunit K
CPE0524	-1.86	1.49E-03		HAD hydrolase, IIB family
CPE2327	-1.86	2.06E-04	*glmS*	glucosamine--fructose-6-phosphate aminotransferase
CPE2386	-1.88	2.27E-04	*rplO*	50S ribosomal protein L15
CPE1908	-1.88	4.16E-03		cob(I)alamin adenosyltransferase
CPE1700	-1.89	1.26E-04	*rpsB*	30S ribosomal protein S2
CPE1069	-1.89	2.42E-04	*fabD*	malonyl CoA-ACP transacylase
CPE1433	-1.90	3.43E-03	*hemB*	delta-aminolevulinic acid dehydratase
CPE2013	-1.90	1.99E-04		hypothetical protein
CPE1532	-1.91	2.18E-04	*sacA*	sucrose-6-P hydrolase
CPE0831	-1.91	2.45E-04	*bgaL*	beta-galactosidase
CPE2192	-1.91	6.17E-04	*atpL*	ATP synthase F0F1 subunit C
CPE1466	-1.91	1.97E-04		PTS system protein
CPE0036	-1.92	7.77E-05	*plc*	phospholipase C
SR2	-1.92	1.92E-04		Region between CPE0013 and CPE0014
SR73	-1.93	6.76E-05		Region between CPE2167 and CPE2168
CPE2190	-1.93	1.99E-04	*atpD*	ATP synthase F0F1 subunit delta
CPE2606	-1.94	6.39E-04	*murA*	UDP-N-acetylglucosamine 1-carboxyvinyltransferase
CPE1526	-1.94	3.57E-04		ABC transporter
SR28	-1.94	1.70E-04	*trnA-met*	Region between CPE0511 and CPE0512
CPE0594	-1.96	1.66E-03		hydrolase, NUDIX family
CPE1538	-1.96	7.13E-03	*asrA*	anaerobic sulfite reductase subunit A

CPE1689	-1.97	3.83E-05	*nusA*	transcription elongation factor NusA
CPE2501	-1.98	1.22E-04		leucyl aminopeptidase
CPE1954	-1.99	9.84E-05		hypothetical protein
PCP55	-2.00	5.92E-04		Spo0A-like protein
CPE1732	-2.01	3.53E-05		DAK2 domain protein
CPE2402	-2.01	1.01E-04	*rplB*	50S ribosomal protein L2
CPE1738	-2.02	4.28E-05		pobable serine/threonine protein kinase
CPE2450	-2.02	3.33E-03		hypothetical protein
CPE2338	-2.04	4.41E-05	*malQ*	4-alpha-glucanotransferase
CPE0854	-2.04	8.07E-03	*aspC*	aminotransferase class V
CPE2660	-2.04	6.25E-05	*rpmH*	50S ribosomal protein L34
CPE0318	-2.05	5.12E-04	*fucI*	L-fucose isomerase
CPE1464	-2.05	8.02E-05		PTS system protein
CPE1394	-2.05	7.28E-05	*tig*	trigger factor
CPE1059	-2.07	7.22E-05		hypothetical protein
CPE1881	-2.09	2.13E-04	*mutS2*	recombination and DNA strand exchange inhibitor protein
CPE0695	-2.09	2.06E-03	*aroB*	3-dehydroquinate synthase
CPE1739	-2.10	2.55E-04		protein phosphatase 2C
CPE1465	-2.10	3.88E-05		PTS system protein
CPE2448	-2.10	3.24E-05		PSP1 domain protein
CPE2230	-2.10	2.40E-05		serine protease
CPE2251	-2.11	1.88E-04	*pepD*	aminoacyl-histidine dipeptidase
CPE2424	-2.11	7.28E-05		RNA methyltransferase, TrmH family, group 3
CPE0091	-2.13	5.92E-04	*iolE*	myo-inositol catabolism protein
CPE0573	-2.14	1.59E-04		hypothetical protein
CPE1208	-2.14	9.61E-05		hypothetical protein
CPE2349	-2.14	3.24E-05		D-amino acid dehydrogenase small subunit
CPE2416	-2.14	7.39E-05	*rplA*	50S ribosomal protein L1
CPE2191	-2.15	3.98E-05	*atpF*	ATP synthase F0F1 subunit B
CPE0319	-2.17	9.74E-05	*fucA*	L-fuculose phosphate aldolase
CPE0442	-2.19	3.86E-04		BFD-like iron-sulfur cluster-binding protein
PCP45	-2.19	1.00E-04		hypothetical protein
CPE0818	-2.19	8.99E-05		endo-beta-N-acetylglucosaminidase
CPE2403	-2.19	3.72E-05	*rplW*	50S ribosomal protein L23
CPE1368	-2.20	3.65E-04		hypothetical protein
CPE2483	-2.22	1.25E-05	*prsA*	peptidylprolyl isomerase
CPE1397	-2.22	8.00E-04		hypothetical protein
CPE2488	-2.22	2.99E-05		DNA-binding response regulator
CPE0144	-2.23	4.03E-03		RNA 2'-phosphotransferase-like protein
CPE2129	-2.23	5.87E-05		hypothetical protein
PCP53	-2.23	4.37E-05		hypothetical protein
CPE1862	-2.24	6.88E-05	*murE*	UDP-N-acetylmuramoylalanyl-D-glutamate--2, 6-diaminopimelate ligase
CPE2417	-2.24	7.02E-05	*rplK*	50S ribosomal protein L11
CPE2130	-2.24	6.79E-05	*rplU*	50S ribosomal protein L21

CPE2466	-2.24	1.29E-04	*greA*	transcription elongation factor GreA
CPE2343	-2.26	1.50E-05		maltose ABC transporter
CPE0739	-2.26	8.20E-03		thioredoxin
CPE2303	-2.27	9.80E-05		heavy metal-transporting ATPase
CPE0732	-2.27	5.39E-04		hypothetical protein
CPE0321	-2.28	4.89E-05		PTS system protein
CPE2289	-2.28	1.13E-05	*groEL*	molecular chaperone GroEL
CPE2404	-2.29	3.24E-05	*rplD*	50S ribosomal protein L4
CPE0297	-2.29	3.24E-05	*tktC*	transketolase C-terminal section
PCP47	-2.31	1.70E-04	*topA*	type I topoisomease
CPE1718	-2.31	5.98E-05	*rnc*	ribonuclease III
CPE1780	-2.31	8.19E-05	*alaS*	alanyl-tRNA synthetase
CPE1861	-2.32	4.41E-05	*murF*	UDP-N-acetylmuramoyl-tripeptide--D-alanyl-D- alanine ligase
CPE0326	-2.32	4.31E-03	*lacA*	galactose-6-phosphate isomerase subunit LacA
CPE1012	-2.33	2.65E-05		endoribonuclease L-PSP
CPE2618	-2.35	9.97E-05		hypothetical protein
CPE1209	-2.36	3.30E-05		hypothetical protein
CPE0381	-2.36	5.16E-04		hypothetical protein
CPE1717	-2.36	3.39E-05		radical SAM domain protein
CPE1876	-2.36	5.71E-04		hypothetical protein
CPE2376	-2.37	3.42E-05	*rpoA*	DNA-directed RNA polymerase subunit alpha
CPE1074	-2.37	3.24E-05	*accC*	biotin carboxylase
CPE2641	-2.37	6.98E-05		phage-related single-strand DNA-binding protein
CPE1698	-2.38	1.53E-05	*pyrH*	uridylate kinase
CPE1044	-2.39	3.16E-04		hypothetical protein
CPE1939	-2.39	1.71E-05	*apt*	adenine phosphoribosyltransferase
CPE2391	-2.40	1.24E-05	*rpsH*	30S ribosomal protein S8
CPE2189	-2.41	7.96E-06	*atpA*	ATP synthase F0F1 subunit alpha
CPE1765	-2.41	8.66E-06	*ftsZ*	cell division protein FtsZ
CPE2188	-2.41	2.40E-05	*atpG*	ATP synthase F0F1 subunit gamma
CPE2052	-2.42	1.29E-05	*deoC*	deoxyribose-phosphate aldolase
CPE1951	-2.42	1.06E-04	*cpdA*	2', 3'-cyclic nucleotide 2'-phosphodiesterase
CPE1354	-2.42	7.16E-06	*entB*	enterotoxin
CPE2187	-2.43	1.14E-05	*atpB*	ATP synthase F0F1 subunit beta
CPE2400	-2.44	2.41E-05	*rplV*	50S ribosomal protein L22
PCP37	-2.45	2.55E-03		hypothetical protein
CPE2434	-2.45	7.74E-06	*nagB*	glucosamine-6-phosphate deaminase
CPE2642	-2.47	3.83E-05	*rpsF*	30S ribosomal protein S6
CPE2652	-2.48	9.00E-05	*soj*	sporulation initiation inhibitor protein soj
PCP46	-2.48	1.70E-04		conjugal transfer ATP-binding protein TraC
CPE1068	-2.48	9.97E-05	*fabH*	3-oxoacyl-ACP synthase
CPE0998	-2.49	8.86E-06	*gltX*	glutamyl-tRNA synthetase
CPE2387	-2.50	2.16E-05	*rpmD*	50S ribosomal protein L30
CPE2405	-2.51	1.24E-05	*rplC*	50S ribosomal protein L3

CPE2447	-2.51	3.42E-05	*fer*	ferredoxin
CPE2301	-2.52	8.02E-06	*crt*	3-hydroxybutyryl-CoA dehydratase
CPE1233	-2.53	9.00E-06		hypothetical protein
CPE0329	-2.53	3.99E-06		hypothetical protein
CPE2408	-2.54	2.47E-05	*fus*	elongation factor G
CPE2530	-2.55	4.48E-06		xaa-pro aminopeptidase
CPE2428	-2.57	2.42E-05	*proS*	prolyl-tRNA synthetase
CPE1472	-2.57	6.50E-06		hypothetical protein
CPE2186	-2.58	1.76E-05	*atpC*	ATP synthase F0F1 subunit epsilon
CPE2414	-2.58	9.59E-06	*rplL*	50S ribosomal protein L7/L12
CPE2415	-2.63	1.25E-05	*rplJ*	50S ribosomal protein L10
CPE0029	-2.63	1.33E-03		TetR family transcriptional regulator
CPE1073	-2.64	3.85E-05	*fabZ*	(3R)-hydroxymyristoyl-ACP dehydratase
CPE0664	-2.64	9.52E-06		hypothetical protein
CPE1321	-2.65	2.64E-04	*cysE*	serine O-acetyltransferase
CPE0296	-2.68	1.14E-05	*tktN*	transketolase N-terminal section
CPE2294	-2.70	3.80E-04		hypothetical protein
CPE1030	-2.70	3.91E-05		hypothetical protein
CPE1072	-2.73	4.32E-05	*accB*	acetyl-CoA carboxylase biotin carboxyl carrier protein
CPE1536	-2.75	1.56E-03	*asrC*	anaerobic sulfite reductase subunit C
CPE0547	-2.76	2.58E-06		nitrite/sulfite reductase-like protein
CPE2377	-2.77	7.37E-06	*rpsD*	30S ribosomal protein S4
CPE2375	-2.78	8.92E-06	*rplQ*	50S ribosomal protein L17
CPE2236	-2.80	1.74E-03		hypothetical protein
CPE0734	-2.81	1.14E-05	*naoX*	pyridine nucleotide-disulphide oxidoreductase
CPE2128	-2.82	9.28E-06	*rpmA*	50S ribosomal protein L27
CPE2016	-2.83	2.70E-06		cytidine deaminase
CPE2379	-2.83	4.17E-06	*rpsM*	30S ribosomal protein S13
CPE1257	-2.83	2.70E-06		hypothetical protein
CPE0576	-2.83	1.71E-05		ABC transporter
CPE2390	-2.84	2.70E-06	*rplF*	50S ribosomal protein L6
PCP44	-2.84	1.37E-04		cell wall-binding protein
CPE2297	-2.84	7.93E-06		3-hydroxybutyryl-CoA dehydrogenase
CPE0014	-2.86	3.42E-06	*serS*	seryl-tRNA synthetase
CPE2392	-2.86	2.70E-06	*rpsN*	30S ribosomal protein S14
CPE0740	-2.89	2.27E-03		hypothetical protein
CPE1231	-2.91	3.81E-06		surface protein
CPE2410	-2.94	6.56E-06	*rpsL*	30S ribosomal protein S12
CPE2298	-2.95	1.15E-05	*fixB*	3-hydroxybutyryl-CoA dehydrogenase
CPE2369	-2.95	1.59E-06	*rpsI*	30S ribosomal protein S9
CPE0421	-2.97	1.41E-03	*glpQ*	glycerophosphodiester phosphodiesterase
CPE1428	-2.97	7.37E-06	*clpB*	clpB protein
CPE1322	-2.98	1.99E-05	*cysK*	cysteine synthase A
CPE2346	-2.99	2.10E-06	*hydA*	iron hydrogenase

CPE0554	-2.99	1.63E-05		hypothetical protein
CPE1523	-3.02	3.05E-06	*nagL*	hyaluronidase
CPE1422	-3.03	4.26E-06		hypothetical protein
CPE1067	-3.06	5.16E-04		MarR family transcriptional regulator
CPE1699	-3.06	1.10E-06	*tsf*	elongation factor Ts
CPE1473	-3.06	2.27E-05		hypothetical protein
CPE2397	-3.08	4.46E-06	*rpmC*	50S ribosomal protein L29
CPE0416	-3.09	8.66E-06	*htpG*	heat shock protein 90
CPE0876	-3.09	8.62E-05		GNAT family acetyltransferase
CPE0530	-3.09	1.26E-05	*ldhD*	D-lactate dehydrogenase
CPE2399	-3.09	3.43E-06	*rpsC*	30S ribosomal protein S3
CPE2304	-3.11	5.28E-04		transcriptional regulator
CPE2393	-3.11	1.24E-06	*rplE*	50S ribosomal protein L5
CPE2290	-3.12	7.39E-04	*groES*	co-chaperonin GroES
CPE2001	-3.12	4.00E-04		radical SAM domain protein
CPE0903	-3.12	3.94E-06	*pduJ*	propanediol utilization protein
CPE2409	-3.14	3.20E-06	*rpsG*	30S ribosomal protein S7
CPE0641	-3.14	8.66E-06	*phoU*	phosphate transporter PhoU
CPE0082	-3.14	4.17E-06		hypothetical protein
CPE0628	-3.16	9.15E-06	*licB*	lic-1 operon protein
CPE2622	-3.16	8.74E-07	*purA*	adenylosuccinate synthetase
CPE2398	-3.17	3.66E-06	*rplP*	50S ribosomal protein L16
CPE2300	-3.18	1.86E-06	*bcd*	butyryl-CoA dehydrogenase
CPE1531	-3.19	7.08E-06		fructokinase
CPE0278	-3.19	1.23E-06		invasion associated secreted endopeptidase
CPE2421	-3.23	2.15E-06	*tuf*	elongation factor Tu
CPE2396	-3.25	2.40E-06	*rpsQ*	30S ribosomal protein S17
CPE2003	-3.26	1.92E-06		nucleoside hydrolase, IUNH family
CPE1534	-3.27	2.47E-05		PTS system, sucrose-specific IIBC component
CPE2407	-3.28	1.92E-06	*tuf*	elongation factor Tu
CPE0627	-3.29	1.37E-06	*licA*	choline kinase
CPE2478	-3.30	8.99E-05		S4 domain protein
CPE0639	-3.32	2.99E-05		phosphate ABC transporter permease
PCP54	-3.35	1.09E-05		hypothetical protein
CPE2394	-3.37	1.09E-06	*rplX*	50S ribosomal protein L24
CPE2388	-3.38	1.73E-06	*rpsE*	30S ribosomal protein S5
CPE2370	-3.39	1.57E-06	*rplM*	50S ribosomal protein L13
CPE0638	-3.41	8.86E-06		phosphate ABC transporter permease
CPE2035	-3.43	1.16E-05	*hrcA*	heat-inducible transcription repressor
CPE1003	-3.43	4.66E-06		hypothetical protein
CPE1421	-3.44	5.10E-07		hypothetical protein
PCP36	-3.45	6.44E-06		hypothetical protein
PCP35	-3.46	1.24E-06		DNA primase
CPE2555	-3.48	2.83E-06		hypothetical protein

CPE0915	-3.50	2.83E-06		NADPH-dependent FMN reductase
CPE1362	-3.50	3.83E-05		dephospho-CoA kinase
CPE0163	-3.58	8.74E-07	*pfoA*	perfringolysin O
CPE1070	-3.62	2.21E-05	*fabG*	3-ketoacyl-ACP reductase
CPE0640	-3.63	1.58E-05		phosphate transporter ATP-binding protein
CPE2061	-3.66	1.24E-06		pyruvate-flavodoxin oxidoreductase
CPE2378	-3.72	7.53E-07	*rpsK*	30S ribosomal protein S11
CPE0845	-3.73	1.24E-06		hypothetical protein
CPE2389	-3.75	8.74E-07	*rplR*	50S ribosomal protein L18
CPE2195	-3.75	7.53E-07	*atoB*	acetyl-CoA acetyltransferase
CPE2032	-3.77	8.40E-07	*dnaJ*	molecular chaperone DnaJ
SR3	-3.80	7.82E-07	*trnA-Ser*	Region between CPE0041 and CPE0042
CPE1071	-3.81	1.24E-06	*fabF*	3-oxoacyl-ACP synthase
CPE0238	-3.81	2.10E-06		hypothetical protein
CPE2348	-3.88	5.10E-07	*ptb*	phosphate butyryltransferase
CPE2299	-3.88	5.10E-07	*etfB*	electron transfer flavoprotein beta subunit
CPE2395	-3.90	5.10E-07	*rplN*	50S ribosomal protein L14
CPE0875	-3.98	4.08E-07		metalloprotease
CPE0629	-4.00	5.10E-07	*licC*	lic-1 operon protein
CPE0846	-4.08	1.64E-07		alpha-clostripain
CPE1031	-4.11	2.86E-06		transcriptional regulator
CPE0637	-4.14	4.46E-06		phosphate ABC transporter phosphate-binding protein
CPE2034	-4.32	2.72E-07	*grpE*	heat shock protein GrpE
CPE2033	-4.50	7.62E-07	*dnaK*	molecular chaperone DnaK
CPE1006	-4.58	7.06E-08		aminopeptidase 1
CPE2347	-4.69	7.06E-08	*buk*	butyrate kinase
CPE0992	-4.75	1.10E-06		oxidoreductase, 2-nitropropane dioxygenase
CPE0170	-4.95	4.18E-08	*arcD*	arginine/ornithine antiporter
CPE0168	-5.00	2.15E-06	*arcA*	arginine deiminase
CPE0636	-5.22	4.18E-08		phosphate ABC transporter phosphate-binding protein
CPE2074	-5.33	1.70E-06		trans-2-enoyl-CoA reductase
CPE0568	-5.70	5.10E-07	*ribA*	riboflavin biosynthesis protein RibA
CPE0566	-6.09	1.09E-06	*ribB*	riboflavin biosynthesis protein RibD
CPE0172	-6.22	2.17E-09	*argR*	arginine repressor ArgR
CPE0569	-6.52	9.35E-08	*ribH*	6,7-dimethyl-8-ribityllumazine synthase
CPE0171	-6.58	9.30E-10	*arcC*	carbamate kinase
CPE0169	-6.63	9.30E-10	*arcB*	ornithine carbamoyltransferase
CPE0567	-6.81	7.53E-07	*risA*	riboflavin synthase subunit alpha
