# Supplementary material for: Concurrent Host-Pathogen Transcriptional Responses in a Clostridium perfringens Murine Myonecrosis Infection
Source: mBio. 2018 Mar 27;9(2):e00473-18. doi: 10.1128/mBio.00473-18 (PMC5874911; doi:10.1128/mBio.00473-18)
Supplement: TABLE S4 [file mbo002183811st4.pdf]

TABLE S4 Differentially expressed regulatory genes.

Locus tag	Gene name	Product	Log_2 fold change (*in vivo vs in vitro*)	FDR
CPE0084	*iolR*	putative transcription repressor	2.16	1.38E-04
CPE0207		putative two-component sensor histidine kinase	2.01	4.47E-03
CPE0235		putative sensor histidine kinase	3.33	1.91E-03
CPE0245		GGDEF/EAL regulatory domain protein	2.96	3.44E-03
CPE0346		putative transcriptional regulator	3.02	3.84E-03
CPE0347		putative transcriptional regulator	3.06	3.43E-03
CPE0370		putative transcriptional regulator	3.47	5.99E-04
CPE0386		putative transcriptional regulator	2.93	3.78E-04
CPE0642	*revR*	orphan response regulator	-0.93	6.44E-02
CPE0789		putative transcriptional regulator	1.54	1.97E-03
CPE0965		GGDEF regulatory domain protein	4.13	3.10E-04
CPE1144		putative transcriptional regulator	2.10	6.07E-04
CPE1283		putative transcriptional regulator	1.98	2.14E-03
CPE1316		putative sensor histidine kinase	1.64	3.21E-03
CPE1446		transcriptional regulator with HTH motif	0.50	1.96E-01
CPE1447		transcriptional regulator with HTH motif	0.74	7.82E-02
CPE1500	*virS*	VirSR two-component sensor histidine kinase	0.24	8.73E-03
CPE1501	*virR*	VirSR two-component response regulator	1.80	7.06E-02
CPE1512	*reeS*	orphan hybrid sensor histidine kinase	0.96	3.10E-02
CPE1560	*agrD*	autoinducer propeptide	0.10	1.53E-02
CPE1561	*agrB*	accessory gene regulator protein B	0.35	3.54E-01
CPE1759	*nrdR*	putative transcriptional regulator	1.68	9.37E-04
CPE1926		putative sensor histidine kinase	2.25	3.24E-04
CPE1987		putative sensor histidine kinase	3.20	2.01E-03
CPE2168	*tex*	toxin Expression protein	-1.28	1.91E-03
CPE2358		putative sigma-L-dependent transcriptional regulator	2.08	1.65E-04
CPE2494		putative transcriptional regulator	1.69	4.77E-04
CPE2539	*ccpA*	putative transcriptional regulator	1.30	4.96E-03
