# Supplementary material for: Concurrent Host-Pathogen Transcriptional Responses in a Clostridium perfringens Murine Myonecrosis Infection
Source: mBio. 2018 Mar 27;9(2):e00473-18. doi: 10.1128/mBio.00473-18 (PMC5874911; doi:10.1128/mBio.00473-18)
Supplement: TABLE S5 [file mbo002183811st5.pdf]

TABLE S5 Differentially expressed pCP13 genes*.

Locus Tag	Log_2 Fold Change	FDR	Gene	Product
PCP12	5.21	3.22E-05		hypothetical protein
PCP10	4.64	2.47E-04		transposase
PCP20	4.16	3.80E-04		hypothetical protein
PCP05	3.89	2.75E-03		transposase
PCP13	3.38	2.44E-03		hypothetical protein
PCP30	2.59	7.25E-03		hypothetical protein
PCP63	2.36	5.69E-05		hypothetical protein
PCP59	2.01	1.89E-04		hypothetical protein
PCP60	1.90	1.66E-04		hypothetical protein
PCP62	1.90	2.80E-03		hypothetical protein
PCP58	1.81	6.17E-04		PemK
PCP33	1.46	2.27E-03		hypothetical protein
PCP32	1.23	5.95E-03		resolvase
PCP57	-1.10	8.22E-03	*cnaB*	collagen adhesin
PCP51	-1.21	6.70E-03		type IV secretion system protein VirD4
PCP50	-1.27	4.56E-03		hypothetical protein
PCP29	-1.37	2.26E-03		hypothetical protein
PCP39	-1.70	3.21E-03		hypothetical protein
PCP55	-2.00	5.92E-04		Spo0A-like protein
PCP45	-2.19	1.00E-04		hypothetical protein
PCP53	-2.23	4.37E-05		hypothetical protein
PCP47	-2.31	1.70E-04	*topA*	type I topoisomease
PCP37	-2.45	2.55E-03		hypothetical protein
PCP46	-2.48	1.70E-04		conjugal transfer ATP-binding protein TraC
PCP44	-2.84	1.37E-04		cell wall-binding protein
PCP54	-3.35	1.09E-05		hypothetical protein
PCP36	-3.45	6.44E-06		hypothetical protein
PCP35	-3.46	1.24E-06		DNA primase

* defined by FDR <0.01 and log_2 (fold change) > 1.
