# Supplementary material for: Concurrent Host-Pathogen Transcriptional Responses in a Clostridium perfringens Murine Myonecrosis Infection
Source: mBio. 2018 Mar 27;9(2):e00473-18. doi: 10.1128/mBio.00473-18 (PMC5874911; doi:10.1128/mBio.00473-18)
Supplement: TABLE S6 [file mbo002183811st6.pdf]

TABLE S6 Summary of mapped *C. perfringens* and murine RNA-seq reads.

	**Mapped reads**	
C. perfringens	***in vivo*** [a]	***in vitro*** [a]
**Replicate 1**	2,164,911	1,819,981
**Replicate 2**	1,823,412	1,389,633
**Replicate 3**	1,759,987	1,374,988
**Total reads**	**5,748,310**	**4,584,602**
**Host**	***C. perfringens* infected host**	**Mock-infected host**
**Replicate 1**	21,241,035	27,713,718
**Replicate 2**	22,033,729	25,463,014
**Replicate 3**	19,942,403	29,983,040
**Total reads**	**63,217,167**	**83,159,772**

[a] RNA-seq analysis of the enriched RNA samples from the *in vivo*- and *in vitro*-derived *C. perfringens* cells was carried out on Illumina Hi-seq and Mi-seq instruments, respectively, which accounts for higher number of reads in the *in vivo* samples. .
