# Supplementary material for: Concurrent Host-Pathogen Transcriptional Responses in a Clostridium perfringens Murine Myonecrosis Infection
Source: mBio. 2018 Mar 27;9(2):e00473-18. doi: 10.1128/mBio.00473-18 (PMC5874911; doi:10.1128/mBio.00473-18)
Supplement: TABLE S7 [file mbo002183811st7.pdf]

TABLE S7 Oligonucleotide primers for QRT-PCR reactions.

Name	Sequence (5’-3’)	Function
**JRP2479**	CCATCTGTTTTTATATCTGCTCCAGTA	*rpoA,* forward primer
**JRP2480**	GGAAGGTGAAGGACCAAAAACTATT	*rpoA,* reverse primer
**JRP5778**	GGGGCAGCTAGCCTCATTA	*pscX,* forward primer
**JRP5779**	CATCGCCTAACCACCAATTT	*pscXF,* reverse primer
**JRP5794**	GCCAATAAAGATGAAATGGGTTT	*fbpA,* forward primer
**JRP5795**	TTTCAGATGCAGGAGGGAAT	*fbpA,* reverse primer
**JRP5922**	TTTTTCCACCACATCAGCAG	*pilT,* forward primer
**JRP5923**	CTCCATCAACGGTTTCCACT	*pilT,* reverse primer
**JRP5780**	TGGCCATATTGATTCCACTG	*risA,* forward primer
**JRP5781**	TTCCCCTTTAACCTCTGCAA	*risA,* reverse primer
**JRP4126**	GGAGCAATTGATGAGTTAGTGTCTAAGT	*pfoA,* forward primer
**JRP4127**	TTCTGAATATTGAGTTCTTGCTGGTAA	*pfoA,* reverse primer
**JRP5406**	TGTAAGGCGCTTATTTGTGC	*plc,* forward primer
**JRP5407**	AAGCGTAGACTTTAGTTGATGCC	*plc,* reverse primer
**JRP5924**	TGTGTTTTTGAAGGGGTAGGA	*codY,* forward primer
**JRP5925**	TCATCATTAAATTCTTCCCCAAA	*codY,* reverse primer
**TLR2F**	CCCTGTGCCACCATTTCC	*Tlr2,* forward primer
**TLR2R**	CCACGCCCACATCATTCTC	*Tlr2,* reverse primer
**NFκBIZF**	TGATGAGGAAGGGAGCTGAC	*Nfκbiz,* forward primer
**NFκBIZR**	TGAATGGACTTCCCCTTCAG	*Nfκbiz,* reverse primer
**TNFF**	CAAATTCGAGTGACAAGCCTG	*Tnf,* forward primer
**TNFR**	GAGATCCATGCCGTTGGC	*Tnf,* reverse primer
**CXCL2F**	AACATCCAGAGCTTGAGTGTGA	*Cxcl2,* forward primer
**CXCL2R**	TTCAGGGTCAAGGCAAACTT	*Cxcl2,* reverse primer
**Il1bF**	CAACCAACAAGTGATATTCTCCATG	*Il1b,* forward primer
**Il1bR**	GATCCACACTCTCCAGCTGCA	*Il1b,* reverse primer
**Il6F**	ATGGATGCTACCAAACTGGAT	*Il6,* forward primer
**Il6R**	TGAAGGACTCTGGCTTTGTCT	*Il6,* reverse primer
